# Quantitative Trait Loci (QTL) Associated with Resistance to a Monogenean Parasite (*Benedenia seriolae*) in Yellowtail (*Seriola quinqueradiata*) through Genome Wide Analysis

**DOI:** 10.1371/journal.pone.0064987

**Published:** 2013-06-04

**Authors:** Akiyuki Ozaki, Kazunori Yoshida, Kanako Fuji, Satoshi Kubota, Wataru Kai, Jun-ya Aoki, Yumi Kawabata, Junpei Suzuki, Kazuki Akita, Takashi Koyama, Masahiro Nakagawa, Takurou Hotta, Tatsuo Tsuzaki, Nobuaki Okamoto, Kazuo Araki, Takashi Sakamoto

**Affiliations:** 1 National Research Institute of Aquaculture, Fisheries Research Agency, Nakatsuhamaura, Minamiise-cho, Watarai-gun, Mie, Japan; 2 Seikai National Fisheries Research Institute, Fisheries Research Agency, Nunoura, Tamanoura-machi, Goto-shi, Nagasaki, Japan; 3 Faculty of Marine Science, Tokyo University of Marine Science and Technology, Konan, Minato-ku, Tokyo, Japan; Pennsylvania State University, United States of America

## Abstract

Benedenia infections caused by the monogenean fluke ectoparasite *Benedenia seriolae* seriously impact marine finfish aquaculture. Genetic variation has been inferred to play a significant role in determining the susceptibility to this parasitic disease. To evaluate the genetic basis of Benedenia disease resistance in yellowtail (*Seriola quinqueradiata*), a genome-wide and chromosome-wide linkage analyses were initiated using F_1_ yellowtail families (n = 90 per family) based on a high-density linkage map with 860 microsatellite and 142 single nucleotide polymorphism (SNP) markers. Two major quantitative trait loci (QTL) regions on linkage groups Squ2 (*BDR-1*) and Squ20 (*BDR-2*) were identified. These QTL regions explained 32.9–35.5% of the phenotypic variance. On the other hand, we investigated the relationship between QTL for susceptibility to *B. seriolae* and QTL for fish body size. The QTL related to growth was found on another linkage group (Squ7). As a result, this is the first genetic evidence that contributes to detailing phenotypic resistance to Benedenia disease, and the results will help resolve the mechanism of resistance to this important parasitic infection of yellowtail.

## Introduction

The production of cultured species of yellowtail in Japan was approximately 152,800 tons in 2009, which accounts for 59% of marine finfish aquaculture in Japan [1]. Yellowtail has been cultured in southern areas of Japan using juveniles caught from natural stock. But in recent years the harvest quantity has declined probably because of decreasing wild populations [2]. Capture-based aquaculture however negatively impacts wild stocks of the targeted species as well as non-targeted species. Therefore, it is expected that artificially produced seed will eventually replace seeds caught from the natural source [3], [4]. Although research on disease, nutrition and pond management has supported the development of the yellowtail aquaculture industry, genetic improvement programs leading to improve yellowtail lines are only at the beginning.

Genetic linkage maps play a prominent role in many areas of genetics, including quantitative trait locus (QTL) analysis, marker-assisted selection (MAS), positional candidate or positional cloning of genes approach, and comparative genomics. The first genetic linkage map for yellowtail was conducted by Ohara et al. [5]. Recently, a second generation map that spans the genome at a higher resolution has been constructed for *Seriola quinqueradiata* [6]. The map contains several hundred markers with microsatellites associated with candidate genes. The map will facilitate genome mapping efforts in *S. quinqueradiata*, and other related species. The mapping data can be compared to reference species and utilized for QTL analyses and further MAS breeding programs of yellowtail.

Benedenia disease caused by infection by *Benedenia seriolae* is a serious parasitic disease for yellowtail in aquaculture, leading to secondary infection due to viral or bacterial disease. This is because fish rub their bodies against the fish cage to remove the parasite, in certain conditions the mortality is quite high especially in juvenile fishes. Although the way of removing the parasite is generally to soak the fish in a freshwater bath, this method requires a high cost and is labor intensive. Thus, Benedenia disease is difficult to prevent in marine aquaculture systems. Besides from the point of view of wildlife conservation, yellowtail aquaculture is considered as a hotbed for enhancement of parasite transmission [7], [8]. Risk management is an important consideration for the long-term sustainability of the aquaculture industry.

*S. quinqueradiata* is generally regarded to have a higher inherent resistance to *B. seriolae* than other kinds of yellowtail, such as *S. lalandi* and *S. dumerili* [9], [10]. The levels of infestation among individuals in *S. quinqueradiata* have been observed to have some degree of heritable variation. These results confirmed earlier evidence of genetic variation to susceptibility to *B. seriolae* in *S. quinqueradiata* and indicate that the host genes play a significant role in determining infection levels against the parasite [9], [10].

Genetic studies of parasitic infections have been reported. A study of *Myxobolus cerebralis* infection in rainbow trout phenotype primarily focused on QTL [11] and *Lepeophtheirus salmonis* in Atlantic salmon phenotype focused on candidate genes [12]. Based on QTL and candidate gene changes in response to infection, these studies have allowed us to gain insights into potential genes and pathways that may be differentially regulated between resistant and susceptible strains. This is an important step towards understanding host responses to infection, however, much remains to be learned about the genetic basis controlling the immunological response to parasitic infection.

In this study, we performed QTL analyses using wild F_1_ strains of *S. quinqueradiata* to elucidate the genetic evidence of resistance to Benedenia disease. By using the high-density linkage map with microsatellite and SNP markers, we identified two major QTL regions contributing to the Benedenia disease resistance. The discovery of a large QTL effect for Benedenia disease resistance has broad implications for improving our general understanding of external parasitic diseases and host pathogen interactions.

## Results

### Phenotypic Trait Correlation with Fish Size and Number of Parasites (*B. seriolae)* in Family A and B

Pearson correlation coefficients for total length, body length, body weight, surface area and number of pathogens are shown in Table 1. Weight and length were positively correlated with each other and, to a lesser degree, the number of pathogens and fish size variables were negatively correlated in family A. However in family B, the number of pathogens was marginally correlated with the fish size variables (*P* = 0.001).

**Table 1:** Between fish size and number of parasites in pairwise Pearson correlations.

Family A		body length	body weight		surface area		number of *B. seriolae*		
	total length	**0.967**	**0.932**		**0.999**		0.050		
	body length		**0.947**		**0.963**		0.041		
	body weight				**0.933**		0.094		
	surface area						0.050		
Family B		body length		body weight		surface area		number of *B. seriolae*	
	total length	**0.964**		**0.933**		**0.999**		**0.389**	
	body length			**0.946**		**0.964**		**0.386**	
	body weight					**0.935**		**0.429**	
	surface area							**0.390**	

Values in bold are different from a significance level *P* = 0.001.

### 1^st^ Screening by Kruskal-Wallis Analysis (K-W test) of Family A for Benedenia Disease Resistance

In analysis of family A, twelve markers were significant (*P<*0.01) on linkage group corresponding to chromosome Squ2, twenty-five markers were significant on linkage group Squ8, thirty-one markers were significant on linkage group Squ20, as 1^st^ screening about Benedenia disease resistance QTL candidates using Kruskal-Wallis analysis (K-W test) (Table 2). A total of sixty-eight markers were informative and indicative of only one family. All of these markers achieved QTL possible using the MapQTL 5 software.

**Table 2:** Significant markers for Benedenia disease resistance using Kruskal–Wallis analysis with A and B families.

		Family A			Family B	
Linkage	Locus	K-W test	Signif.	Linkage	K-W test	Signif.
group				group		
Squ2F	Sequ0941TUF	0.005	NS	Squ2M	2.772	*
	Sequ0020TUF	0.005	NS		2.893	*
	Sequ2216BAC	0.005	NS		3.403	*
	Sequ0603TUF	0.041	NS		4.106	**
	Sequ0832TUF	0.041	NS		2.743	*
	Sequ0648TUF	0.041	NS		3.725	*
	Sequ3121BAC	0.041	NS		3.725	*
	Sequ0174TUF	0.249	NS		3.725	*
	Sequ0171BAC	6.901	***		3.725	*
	Sequ0172TUF	8.966	****		3.725	*
	Sequ0672TUF	12.926	******		3.725	*
	Sequ0125TUF	16.83	*******		3.725	*
	Sequ1065TUF	16.83	*******		–	
	Sequ1295BAC	18.998	*******		–	
	Sequ1066TUF	17.478	*******		–	
	Sequ0979BAC	17.478	*******		3.725	*
	Sequ1067TUF	14.393	******		–	
	Sequ1068TUF	7.961	****		–	
	Sequ1069TUF	9.115	****		–	
	Sequ1070TUF	7.136	***		–	
	Sequ0788TUF	5.404	**		3.76	*
	Sequ0549TUF	2.939	*		3.725	*
	Sequ1026TUF	2.939	*		–	
	Sequ1749BAC	2.939	*		3.725	*
	Sequ0827TUF	2.866	*		3.725	*
	Sequ0828TUF	2.866	*		3.725	*
Squ8F	Sequ0074TUF	9.852	****	Squ8M	0	NS
	Sequ0409TUF	9.852	****		0.728	NS
	Sequ0431TUF	9.852	****		0.322	NS
	Sequ0851TUF	9.852	****		0.605	NS
	Sequ0906TUF	9.852	****		0.728	NS
	Sequ3175BAC	9.852	****		0.103	NS
	Sequ2078BAC	9.852	****		0.911	NS
	Sequ1769BAC	10.247	****		0.029	NS
	Sequ0670BAC	10.398	****		0.064	NS
	Sequ0503TUF	10.398	****		0.485	NS
	Sequ0610TUF	10.398	****		–	
	Sequ0101TUF	10.398	****		1.804	NS
	Sequ2536BAC	10.398	****		1.804	NS
	Sequ2198BAC	10.398	****		3.506	*
	Sequ0985TUF	10.398	****		1.973	NS
	Sequ1013TUF	10.398	****		1.973	NS
	Sequ0507BAC	10.398	****		–	
	Sequ0575BAC	10.398	****		–	
	Sequ2218BAC	10.398	****		1.973	NS
	Sequ3193BAC	10.398	****		–	
	Sequ3288BAC	10.398	****		–	
	Sequ00955SNP	10.398	****		–	
	Sequ01036SNP	10.398	****		–	
	Sequ02608SNP	10.398	****		–	
	Sequ02777SNP	10.398	****		–	
Squ20F	Sequ1071TUF	12.821	******	Squ20M	–	
	Sequ0439TUF	12.821	******		2.721	*
	Sequ2134BAC	12.821	******		–	
	Sequ1100BAC	12.821	******		0.808	NS
	Sequ01056SNP	12.038	*****		–	
	Sequ02679SNP	12.821	******		–	
	Sequ1072TUF	11.438	*****		–	
	Sequ3071BAC	11.438	*****		8.263	****
	Sequ0938TUF	11.438	*****		9.786	****
	Sequ0719TUF	9.914	****		–	
	Sequ1074TUF	9.914	****		–	
	Sequ1075TUF	9.914	****		–	
	Sequ00695SNP	10.844	*****		–	
	Sequ02734SNP	9.371	****		–	
	Sequ2569BAC	9.914	****		8.802	****
	Sequ1073TUF	9.914	****		–	
	Sequ2645BAC	9.914	****		3.928	**
	Sequ1076TUF	9.914	****		–	
	Sequ0537TUF	9.914	****		7.914	****
	Sequ0596BAC	9.914	****		9.011	****
	Sequ0829TUF	9.914	****		–	
	Sequ0836TUF	9.914	****		8.338	****
	Sequ1989BAC	9.914	****		7.161	***
	Sequ2312BAC	9.914	****		–	
	Sequ0017BAC	9.914	****		6.647	***
	Sequ0730TUF	9.914	****		6.647	***
	Sequ1077TUF	10.211	****		–	
	Sequ1078TUF	11.544	*****		–	
	Sequ0808TUF	13.494	******		6.647	***
	Sequ1079TUF	13.494	******		–	
	Sequ0288TUF	10.037	****		6.647	***
	Sequ1702BAC	0.036	NS		6.647	***

Signif.; Significance levels:

* <0.1

** <0.05

*** <0.01

**** <0.005

***** <0.001

****** <0.0005

******* <0.0001.

NS; not significant, -; not informative in this locus.

Squ(linkage group)F; F is dam allele in female linkage group. Squ(linkage group)M; M is sire allele in male linkage group.

### Simple Interval Mapping Results about All Linkage Groups in Family A

**Figure 1:**
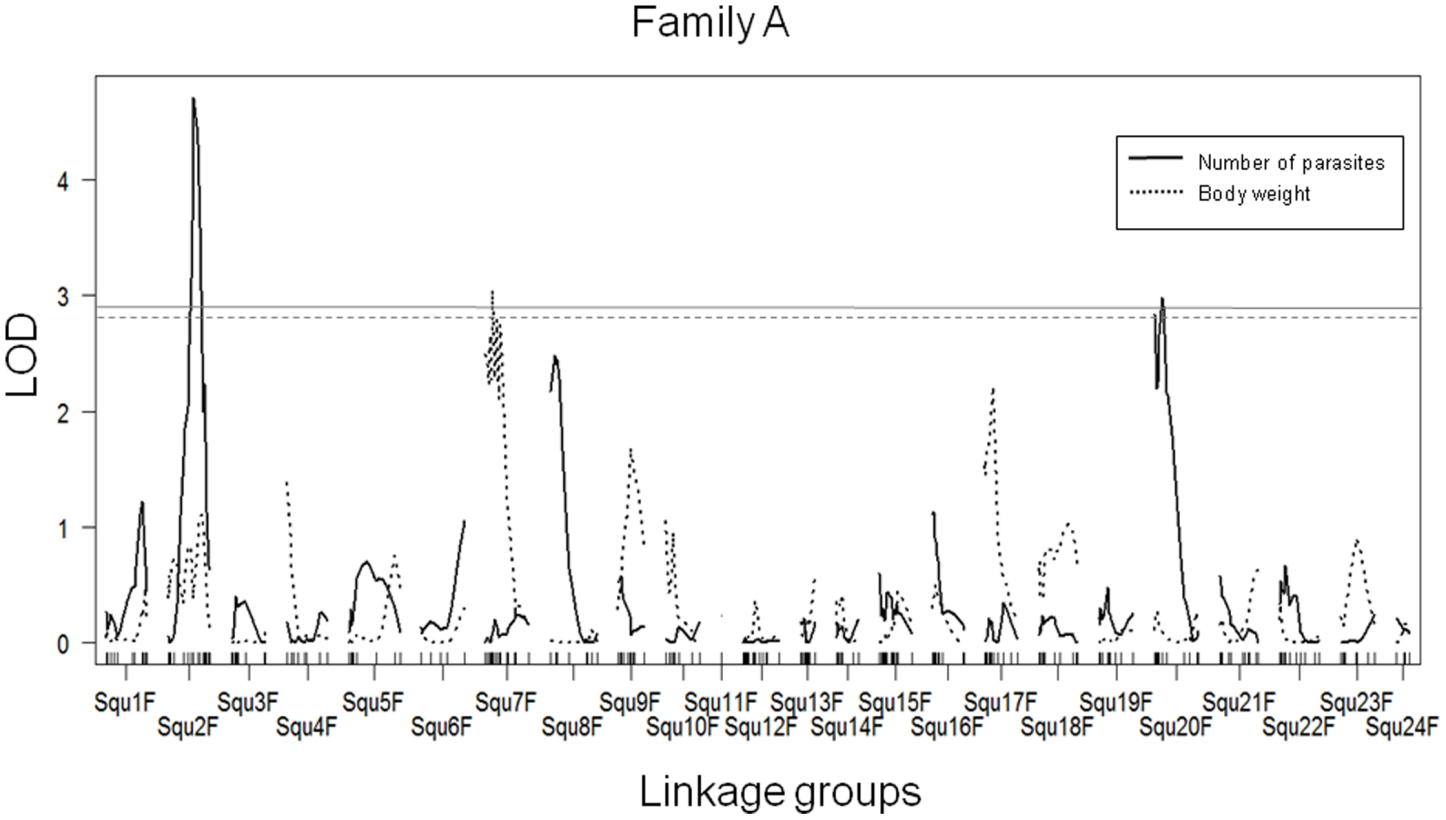
Simple interval mapping results for Benedenia disease resistance and body weight in all linkage groups with family A. Squ(linkage group)F; marker distance in female map. This figure is described using R/qtl. Number of parasites; *Pg* <0.05 significant threshold is indicated as solid line. Body weight; *Pg* <0.05 significant is indicated as dashed line.

**Figure 2:**
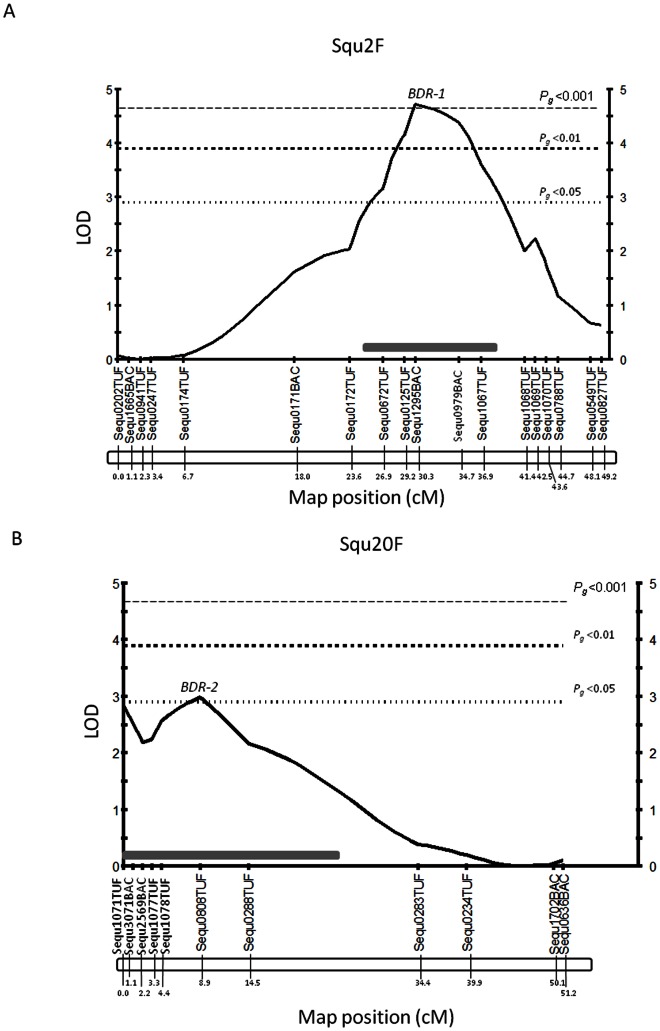
Localization of significant markers for Benedenia disease resistance in linkage group Squ2F and Squ20F with family A. Squ(linkage group)F; marker distance in female map. (A) Squ2F, (B) Squ20F. Map positions and LOD scores are based on a simple interval mapping QTL analysis using the software MapQTL 5. Marker absolute map distances are given in (cM). 95% confidence probability LOD support interval was indicated as Gray bold line. Horizontal lines across each plot indicate LOD siginificance threshold, *P_g_*; genome-wide significance threshold.

We show the interval mapping results for Benedenia disease resistance in family A for all linkage groups in Figure 1. Three regions of the chromosomes were identified to be significantly associated with Benedenia desease for family A (Table 3). The QTL at Squ2 identified by simple interval mapping were also found by using K-W test. The peak LOD value of sequ1295BAC (LOD = 4.71) was substantially higher than the genome-wide LOD significance threshold value of 2.9 determined by permutation testing (*Pg* <0.05; Pg: P value genome-wide LOD). Linkage group in Squ2 QTL region (tentatively termed *BDR-1*) was observed as a high single peak as genome-wide LOD significance level (*Pg* <0.001) in interval mapping (Figure 2A). The markers of chromosomal region of Squ8 linkage group, example sequ0670BAC (LOD = 2.45), was less than the genome-wide LOD significance level (*Pg* <0.05). The markers of chromosomal region of Squ20 linkage group, example Sequ0808TUF (LOD = 2.98), had slightly exceeded the genome-wide LOD significance level (*Pg* <0.05) (Figure 2B). About one of the peaks, it can tentatively be called as the *BDR-2* significant region, based on the rules of QTL nomenclature [13], [14].

**Table 3:** Simple interval mapping results of the significant markers for Benedenia disease resistance in linkage group 2, 8, and 20 with two families.

		Family A				Family B		
Linkage Group	Locus	LOD	% Var.	Effect	Linkage Group	LOD	% Var.	Effect
Squ2F	Sequ0171BAC	1.61	7.9	0.60	Squ2M	NS		
	Sequ0172TUF	2.04	9.9	0.66		NS		
	Sequ0672TUF	3.15	14.9	0.82		NS		
	Sequ0125TUF	4.15	19.1	0.94		NS		
	Sequ1065TUF	4.15	19.1	0.94		NS		
	Sequ1295BAC	**4.71**	**21.4**	**1.00**		NS		
	Sequ1066TUF	4.38	20.1	0.96		NS		
	Sequ0979BAC	4.38	20.1	0.96		NS		
	Sequ1067TUF	3.60	16.8	0.89		NS		
	Sequ1068TUF	2.00	9.7	0.66		NS		
	Sequ1069TUF	2.24	10.8	0.70		NS		
	Sequ1070TUF	1.74	8.5	0.62		NS		
	Sequ0788TUF	1.17	5.8	0.51		NS		
Squ8F	Sequ0074TUF	2.17	10.5	0.68	Squ8M	NS		
	Sequ0409TUF	2.17	10.5	0.68		NS		
	Sequ0431TUF	2.17	10.5	0.68		NS		
	Sequ0851TUF	2.17	10.5	0.68		NS		
	Sequ0906TUF	2.17	10.5	0.68		NS		
	Sequ3175BAC	2.17	10.5	0.68		NS		
	Sequ2078BAC	2.17	10.5	0.68		NS		
	Sequ1769BAC	2.41	11.6	0.72		NS		
	Sequ0670BAC	**2.45**	**11.8**	**0.72**		NS		
	Sequ0503TUF	**2.45**	**11.8**	**0.72**		NS		
	Sequ0610TUF	**2.45**	**11.8**	**0.72**		NS		
	Sequ0101TUF	**2.45**	**11.8**	**0.72**		NS		
	Sequ2536BAC	**2.45**	**11.8**	**0.72**		NS		
	Sequ2198BAC	**2.45**	**11.8**	**0.72**		NS		
	Sequ0985TUF	**2.45**	**11.8**	**0.72**		NS		
	Sequ1013TUF	**2.45**	**11.8**	**0.72**		NS		
	Sequ0507BAC	**2.45**	**11.8**	**0.72**		NS		
	Sequ0575BAC	**2.45**	**11.8**	**0.72**		NS		
	Sequ2218BAC	**2.45**	**11.8**	**0.72**		NS		
	Sequ3193BAC	**2.45**	**11.8**	**0.72**		NS		
	Sequ3288BAC	**2.45**	**11.8**	**0.72**		NS		
	Sequ00955SNP	**2.45**	**11.8**	**0.72**		NS		
	Sequ01036SNP	**2.45**	**11.8**	**0.72**		NS		
	Sequ02608SNP	**2.45**	**11.8**	**0.72**		NS		
	Sequ02777SNP	**2.45**	**11.8**	**0.72**		NS		
Squ20F	Sequ1071TUF	**2.83**	**13.5**	**0.77**	Squ20M	NS		
	Sequ0439TUF	**2.83**	**13.5**	**0.77**		NS		
	Sequ2134BAC	**2.83**	**13.5**	**0.77**		NS		
	Sequ1100BAC	**2.83**	**13.5**	**0.77**		NS		
	Sequ01056SNP	**2.83**	**13.5**	**0.77**		NS		
	Sequ02679SNP	**2.83**	**13.5**	**0.77**		NS		
	Sequ1072TUF	2.51	12.0	0.73		1.91	9.0	0.61
	Sequ3071BAC	2.51	12.0	0.73		**2.24**	**10.5**	**0.66**
	Sequ0938TUF	2.51	12.0	0.73		**2.24**	**10.5**	**0.66**
	Sequ0719TUF	2.19	10.6	0.68		1.96	9.3	0.62
	Sequ1074TUF	2.19	10.6	0.68		1.96	9.3	0.62
	Sequ1075TUF	2.19	10.6	0.68		1.96	9.3	0.62
	Sequ00695SNP	2.19	10.6	0.68		1.96	9.3	0.62
	Sequ02734SNP	2.19	10.6	0.68		1.96	9.3	0.62
	Sequ2569BAC	2.19	10.6	0.68		1.96	9.3	0.62
	Sequ1073TUF	2.19	10.6	0.68		1.96	9.3	0.62
	Sequ2645BAC	2.19	10.6	0.68		NS		
	Sequ1076TUF	2.19	10.6	0.68		NS		
	Sequ0537TUF	2.19	10.6	0.68		1.66	7.9	0.57
	Sequ0596BAC	2.19	10.6	0.68		**1.91**	**9.0**	**0.61**
	Sequ0829TUF	2.19	10.6	0.68		**1.91**	**9.0**	**0.61**
	Sequ0836TUF	2.19	10.6	0.68		**1.91**	**9.0**	**0.61**
	Sequ1989BAC	2.19	10.6	0.68		1.48	7.1	0.54
	Sequ2312BAC	2.19	10.6	0.68		1.48	7.1	0.54
	Sequ0017BAC	2.19	10.6	0.68		1.39	6.6	0.53
	Sequ0730TUF	2.19	10.6	0.68		1.39	6.6	0.53
	Sequ1077TUF	2.25	10.9	0.69		1.39	6.7	0.53
	Sequ1078TUF	2.56	12.3	0.74		1.39	6.7	0.53
	Sequ0808TUF	**2.98**	**14.1**	**0.79**		1.39	6.6	0.53
	Sequ1079TUF	**2.98**	**14.1**	**0.79**		1.39	6.6	0.53
	Sequ0288TUF	2.17	10.5	0.68		1.39	6.6	0.53

Locus; marker name, LOD; Lod scores, % Var; percent of variance explained, Effect; estimated effect, NS; not significant. Squ(linkage group)F; F is dam allele in female linkage group. Squ(linkage group)M; M is sire allele in male linkage group. Values in bold are LOD max in peak of each QTL marker position and each value.

Each of the LOD peaks, Squ2 (Sequ1295BAC), Squ8 (Sequ0670BAC), Squ20 (Sequ1071TUF), and Squ20 (Sequ0808TUF), can explain the phenotypic variance ranging from 11.8 to 21.4% by simple interval mapping. When LOD peaks were combined into simple interval mapping results, two loci (Sequ1295BAC; Squ2, Sequ0808T TUF; Squ20) could explain the phenotypic variance ranging up to 35.5%. If the other LOD peak of Squ20 (Sequ1071TUF) and the LOD peak of Squ8 (Sequ0670BAC) are added, these four loci were responsible for a significant portion 60.8% of the total phenotypic variation in family A.

### Multiple QTL Model Mapping about Significant Loci in Linkage Groups Squ2 and Squ20

**Figure 3:**
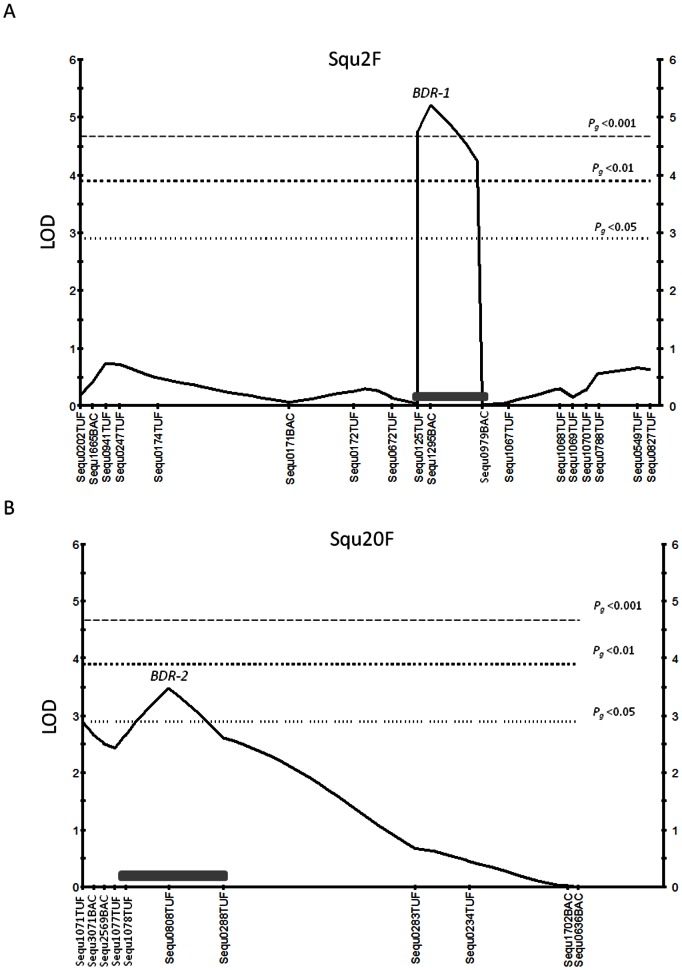
Significant markers for Benedenia disease resistance multiple-QTL model mapping in linkage group Squ2F and Squ20F with family A. Squ(linkage group)F; marker distance in female map. (A) Squ2F, (B) Squ20F. This figure was described using by MapQTL 5.

After simple interval mapping consideration of analysis results of family A, Multiple QTL model was performed. Multiple QTL model mapping was applied to detect significant loci with the exception of ghost QTL, and is based on backward elimination. Therefore significant regions in linkage group Squ8 (ex. Sequ0670BAC) were rejected as QTL region in this step. Map positions and LOD scores are based on multiple QTL model analysis using the software MapQTL 5. The results of the multiple QTL model mapping are shown in Table 4 and Figure 3. Peaks of LOD score were higher than the simple interval mapping results, example *BDR-1* on Squ2 was indicated as LOD = 5.21, and *BDR-2* on Squ20 was indicated as LOD = 3.47. But the marker locus Sequ1071TUF (LOD = 2.89) was less than the genome-wide LOD significance level (*Pg* <0.05) in edge of Squ20 linkage group. LOD peaks were combined into multiple QTL model mapping results, the two loci (Sequ1295BAC; Squ2, Sequ0808T TUF; Squ20) could explain phenotypic variance ranging up to 32.9%.

**Table 4:** Multiple QTL model mapping results of the significant markers for Benedenia disease resistance in linkage group 2 and 20 in family A.

		Family A		
Linkage Group	Locus	LOD	% Var.	Effect
Squ2F	Sequ1065TUF	4.75	18.5	0.92
	Sequ1295BAC	**5.21**	**20.1**	**0.97**
Squ20F	Sequ1071TUF	**2.89**	**10.8**	**0.69**
	Sequ1072TUF	2.66	10.0	0.66
	Sequ3071BAC	2.66	10.0	0.66
	Sequ0938TUF	2.66	10.0	0.66
	Sequ0719TUF	2.50	9.4	0.64
	Sequ1074TUF	2.50	9.4	0.64
	Sequ1075TUF	2.50	9.4	0.64
	Sequ00695SNP	2.50	9.4	0.64
	Sequ02734SNP	2.50	9.4	0.64
	Sequ2569BAC	2.50	9.4	0.64
	Sequ1073TUF	2.50	9.4	0.64
	Sequ2645BAC	2.50	9.4	0.64
	Sequ1076TUF	2.50	9.4	0.64
	Sequ0537TUF	2.50	9.4	0.64
	Sequ0596BAC	2.50	9.4	0.64
	Sequ0829TUF	2.50	9.4	0.64
	Sequ0836TUF	2.50	9.4	0.64
	Sequ1989BAC	2.50	9.4	0.64
	Sequ2312BAC	2.50	9.4	0.64
	Sequ0017BAC	2.50	9.4	0.64
	Sequ0730TUF	2.50	9.4	0.64
	Sequ1077TUF	2.43	9.2	0.64
	Sequ1078TUF	2.66	10.0	0.67
	Sequ0808TUF	**3.47**	**12.8**	**0.75**
	Sequ1079TUF	3.47	12.8	0.75
	Sequ0288TUF	2.61	9.8	0.66

Locus; marker name, LOD; Lod scores, % Var; percent of variance explained, Effect; estimated effect. Squ(linkage group)F; F is dam allele in female linkage group. Values in bold are LOD max in peak of each QTL marker position and each value.

### K-W test of Benedenia disease resistance in family B

About candidate marker loci in family A, we collected genotype data in family B. A total of six markers of linkage group Squ20 showed consistent significant results (*P<*0.005) (Table 2). But one markers of linkage group Squ2 (Sequ0603TUF) were marginally significant (*P<*0.05), while all markers of linkage group Squ8 were not significant (*P<*0.05) in K–W test.

### Simple Interval Mapping Results on Chromosomal-wide Analysis in Family B

After K-W test in family B, simple interval mapping was performed to identify the location of significant QTL regions on Squ2, Squ8, and Squ20 (Table 3). About the candidate QTL region in Squ20 linkage group, loci of which were confirmed to have a significant value. Also these loci were observed to have a significant LOD score 2.24 in family B, which was confirmed in reproducible families chromosome-wide LOD significance level (*Pc* <0.05, Pc: P value chromosome-wide LOD) by interval mapping (Figure S1). Results for both A and B families as two peaks about significant region in linkage group Squ20.

### Linkage Analysis Estimation of Other Phenotype Fish Size QTL Regions

We show the interval mapping for body weight QTL in family A about all linkage groups in Figure 1. Significant loci about fish size (total length, body length, body weight, surface area) in linkage groups Squ7, Squ17 are shown in Table 5. The markers of chromosomal region of Squ7 linkage group, for example body weight, Sequ0582TUF (LOD = 3.04) had values exceeding the genome-wide LOD significance level (LOD 2.8, *Pg* <0.05) in family A (Figure S2). The region of LOD maximum locus (Sequ0582TUF) could explain phenotypic variance ranging 14.4% of the trait body weight. Furthermore in family B analysis about fish size, the region of Squ7 linkage group was significant (*P*<0.05) by K-W test (data not shown). For the fish size QTL candidate region in these families, the number of pathogens was negatively correlated with fish size, the same as the correlation coefficient results in this study.

**Table 5:** Simple interval mapping results of the fish sizes in linkage group 7 and 17 in family A.

		total length			body length			total weight			surface area		
Linkage Group	Locus	LOD	% Var.	Effect	LOD	% Var.	Effect	LOD	% Var.	Effect	LOD	% Var.	Effect
Squ7F	Sequ1041TUF	2.46	11.8	0.70	2.62	12.6	0.72	2.50	12.0	0.70	2.46	11.8	0.70
	Sequ1041BAC	2.65	12.7	0.73	2.67	12.8	0.73	2.43	11.7	0.70	2.65	12.7	0.73
	Sequ3208BAC	2.06	10.0	0.65	2.50	12.0	0.72	2.24	10.8	0.68	2.06	10.0	0.65
	Sequ0447TUF	2.48	11.9	0.71	2.90	13.8	0.77	2.66	12.7	0.73	2.48	11.9	0.71
	Sequ0582TUF	**2.57**	**12.3**	**0.72**	**2.92**	**13.9**	**0.76**	**3.04**	**14.4**	**0.77**	**2.57**	**12.3**	**0.72**
	Sequ0623TUF	2.12	10.3	0.65	2.22	10.7	0.67	2.27	11.0	0.67	2.12	10.3	0.65
	Sequ2990BAC	2.50	12.0	0.70	2.62	12.5	0.72	2.78	13.3	0.74	2.50	12.0	0.70
	Sequ00662SNP	1.83	8.9	0.60	2.02	9.8	0.63	2.10	10.2	0.64	1.83	8.9	0.60
	Sequ0416TUF	2.49	12.0	0.70	2.72	13.0	0.73	2.74	13.1	0.73	2.49	12.0	0.70
	Sequ0784TUF	1.39	6.8	0.53	1.62	7.9	0.57	1.22	6.1	0.50	1.39	6.8	0.53
	Sequ1421BAC	1.23	6.1	0.50	1.47	7.2	0.54	1.15	5.7	0.48	1.23	6.1	0.50
	Sequ0382BAC	0.65	3.3	0.36	0.81	4.1	0.41	0.48	2.5	0.31	0.65	3.3	0.36
	Sequ1628BAC	0.44	2.2	0.30	0.53	2.7	0.33	0.27	1.4	0.24	0.44	2.2	0.30
	Sequ00355_1SNP	0.52	2.6	0.33	0.66	3.3	0.37	0.34	1.7	0.26	0.52	2.6	0.33
	Sequ1700BAC	0.44	2.2	0.30	0.45	2.3	0.30	0.15	0.8	0.18	0.44	2.2	0.30
Squ17F	Sequ1016TUF	1.30	6.4	0.51	1.84	9.0	0.61	1.53	7.6	0.56	1.30	6.4	0.51
	Sequ0025TUF	1.10	5.5	0.47	1.68	8.2	0.58	1.46	7.2	0.54	1.10	5.5	0.47
	Sequ0895TUF	1.30	6.4	0.51	1.82	8.9	0.60	1.68	8.3	0.58	1.30	6.4	0.51
	Sequ0228TUF	1.35	6.7	0.52	1.78	8.7	0.60	1.70	8.3	0.58	1.35	6.7	0.52
	Sequ3088BAC	1.59	7.8	0.56	1.94	9.4	0.62	1.78	8.7	0.59	1.59	7.8	0.56
	Sequ1690BAC	1.73	8.5	0.59	2.15	10.4	0.65	2.01	9.8	0.63	1.73	8.5	0.59
	Sequ01964SNP	**1.79**	**8.8**	**0.60**	**2.30**	**11.1**	**0.67**	**2.22**	**10.8**	**0.66**	**1.79**	**8.8**	**0.60**
	Sequ0716TUF	0.86	4.3	0.42	1.05	5.2	0.46	0.92	4.6	0.43	0.86	4.3	0.42
	Sequ2051BAC	0.45	2.3	0.30	0.60	3.0	0.35	0.56	2.8	0.34	0.45	2.3	0.30
	Sequ2269BAC	0.12	0.6	0.16	0.18	0.9	0.20	0.34	1.7	0.27	0.12	0.6	0.16
	Sequ2942BAC	0.02	0.1	0.06	0.02	0.1	0.06	0.16	0.8	0.18	0.02	0.1	0.06

Locus; marker name, LOD; Lod scores, % Var; percent of variance explained. Effect; estimated effect. Squ(linkage group)F; F is dam allele in female linkage group. Values in bold are LOD max in peak of each QTL, marker position and each value.

## Discussion

This study is the first to report the detection and positioning of major QTLs affecting resistance to external parasites in yellowtail. We identified in yellowtail two chromosomal regions containing QTL (*BDR-1, BDR-2*) that are associated with Benedenia disease resistance. Two putative QTL associations, of intermediate to large effect on Benedenia disease resistance, were localized to linkage groups Squ2 and Squ20.

On Squ2, the example marker loci Sequ1295BAC, which can explain phenotypic variance ranging from 20.1 to 21.4% by simple interval and multiple model interval mapping results. On Squ20, the example marker loci Sequ0808TUF, which can explain phenotypic variance ranging from 12.8 to 14.1% by both mapping methods. These two loci were responsible for a range from 32.9 to 35.5% of the total phenotypic variation in family A. If other peaks which the marker Sequ1071TUF on Squ20 can be considered as significant loci, it will explain the phenotypic variance range from 10.8% to 13.5%. In addition, suggested level marker Sequ0670BAC on Squ8 potentially explains about 11.8% of the phenotypic variance. In total, these four loci can explain phenotypic variance ranging from 55.5% to 60.8% in family A. However, these two additional QTL were only significant at the chromosome-wide level and should be regarded as tentative until other family results are confirmed. Besides QTL interaction was found not to occur between Squ2 region and Squ20 region when considered from the 2D-QTL scan function in R/qtl in family A (data not shown).

Family B was not used in the main genome-wide family analysis. The number of pathogens was marginally significantly correlated with fish size. The family B was analyzed in limited linkage groups with chromosome-wide significance levels to confirm that the candidate QTL regions are reproducible. For the candidate QTL regions in the Squ20 linkage group, which were confirmed to have a significant value in family B. However the highly significant region in family A on linkage group Squ2 was rejected as in family B. Also the suggested region in family A on linkage group Squ8 was rejected in family B. We consider that the reasons for these differences are due to the different parental fish of family B that were selected, and their F_1_ progeny showed more susceptibility to the parasite than that of family A (Figure S3).

The most important finding of this study was detected as a single peak of QTL (*BDR-1*) associated with Benedenia infection resistance within the proximal region of linkage group Squ2. The QTL peak (Sequ1295BAC) was located at position 30.3 cM, with a 95% confidence interval that the QTL region lies within 10 cM of the most proximal marker from Sequ0672TUF to Sequ1067TUF by simple interval mapping. Furthermore the results of multiple QTL model mapping indicated the QTL region within 5.5 cM interval (Figure 3A). The 5.5 cM interval is narrow as QTL candidate region and should be considered as a fine approximation, given the large QTL effect and high recombination rate found in yellowtail females. Besides the QTL peak marker Sequ1295BAC isolated from BAC library end sequencing [6], and adjacent markers Sequ0979BAC were also isolated from BAC library end sequencing. These physical sequences based on BAC library were developed for future identification of positional candidate genes or positional cloning regarding external parasitism disease resistant genes. However before initiative sequences of contignation from BAC library, it would be beneficial to further refine the QTL region by increasing the marker density around the QTL peak, likely with EST based SNP markers. Furthermore it would be necessary to map additional families using near-isogenic lines, which are separated from other QTL effects on other linkage groups, and successive generations with larger progeny sizes to increase the total recombination events. Further QTL studies should therefore focus on fine-mapping the QTL identified on Squ2 as well as searching for additional QTL on other linkage groups in yellowtail.

On the other hand, the QTL (*BDR-2*) significant region would exist as multiple QTL on linkage group Squ20 (Figure 2B, Figure 3B). This is because results of analyses of family A and family B were detected as two peaks. In addition, this QTL significant region was confirmed for Benedenia disease resistance in hybrid lines between *S. quinqueradiata* and *S. lalandi* [15]. Therefore, it is possible that this linkage group is enriched for external parasitism disease resistance genes, although without a sequenced genome, this remains highly speculative. Genetic variation will contribute to the Benedenia disease resistance phenotype in different families or cross species results. Finding this QTL region strongly supports the potential for success of marker-assisted selection (MAS) for this disease. But it is difficult to evaluate each separate QTL effect from these two peaks. Utilization of this QTL has to pay attention to the broad region on Squ20, which should be integrated into the next generation for MAS.

There is another possibility about the identification of the positional candidate gene or utilization for MAS. The synteny study among vertebrate fish species, medaka, zebra, pufferfish, and yellowtail can be considered. The physical BAC clones and EST based SNPs will help to gain new information about both QTLs (*BDR-1, BDR-2*). Still due to the insufficient data about the yellowtail genome, it is difficult to compare the orthologus region or gene based on homological sequence. This approach is now progressing. Some studies on salmonid species have shown significant regions of disease resistance are common in separated genetic research or ortholog regions in cross-species [11], [16], [17], [18], [19], [20]. These research results indicate the necessity of synteny analysis in fish species to determine the orthologous candidate gene about disease resistance. Also the immune-related genes on the same linkage group are correlated with significant QTL about disease resistance. Especially some studies have reported that QTLs are located on the same linkage group with the MHC class I, II or toll-like receptor regions [12], [21], [22], [23], [24]. Linkage groups on QTL region were specifically targeted in these studies because they carry the classical MHC class I and II genes previously linked to differences in susceptibility to viral, bacterial, and parasitic diseases.

Effect of fish size in relation to the number of *B. seriolae*, our results suggest that fish size is not responsible for the resistance of *B. seriolae* in yellowtail, because the number of pathogens and fish size were negatively correlated in family A (Table 1). Although we cannot exclude the possibility that multiple fish growth loci are present within the currently identified *B. seriolae* resistance QTL region in Squ2, and Squ20, the results of significant loci about fish size (total length, body length, body weight, surface area) were identified in different linkage groups in Squ7 by the results of the three month old stage. The results from our current study suggest a negative correlation between growth traits and the number of parasites. However, given the effect of the discovered QTL region has on the Benedenia disease phenotype, it is unlikely that growth has a major role in conferring parasite disease resistance. In the case of whirling disease, caused by the pathogen *Myxobolus cerebralis*, fish age and size were found to be key factors influencing the severity of whirling disease in experimentally infected rainbow trout [25], [26]. In this disease, rainbow trout growing at faster rates may more rapidly become resistant to both clinical symptoms of whirling disease and high numbers of myxospores in their skeletal elements. But our QTL study targeted external parasitism, and as such the case of whirling disease is difficult to compare with Benedenia disease. Fish size QTLs were identified for different linkage groups in Squ7. However, this QTL possibly affects significant locus at the genome-wide level (*P*<0.05) by simple interval mapping results in the three month old stage, about 185 mm size and 65 g weight. For determining growth QTL, it might be necessary to measure the phenotype using different development stages by time series analysis.

Sex was not directly correlated with the number of *B. seriolae* in the fish used in this study. Actually we could not morphologically distinguish males and females at the three month old stage. But sex-linked markers have already been identified on the linkage group Squ12 within several families [27]. Our results indicated that there are no differences between males and females. And we can support the QTL study results about *Lepeophtheirus salmonis* in Atlantic salmon [12]. Hence, no differences were observed between males and females, although fish size, which is known to vary between sexes has been found to be an important factor in determining lice abundance [28].

There are no previous studies that investigated whether host genes are important determinants of susceptibility to *B. seriolae* in yellowtail. However, further research is required to elucidate the functional basis of the differential susceptibility to infection among individuals. Possible mechanisms include a variety of host factors, such as specific and non-specific immune-related genes [29], and skin epithelial extract biochemistry [30]. Perhaps the most significant instances are the inflammatory differences associated with variation in susceptibility to initial infection among yellowtail species [9], [10]. For example, Japanese flounder, exhibit a peripheral blood leucocyte response to the presence of *Neoheterobothrium hirame*, which has been linked to the earlier onset and significant greater up- and down-regulation of pro-inflammatory genes, such as matrix metalloproteinase MMP-9, MMP-13, leukotriene B4 receptor, CD20 receptor, major histocompatibility complex MHC Class I, MHC Class II beta-chain, immunoglobulin light chain and immunoglobulin heavy chain and unknown genes using microarray analysis [29]. In yellowtail, there is a possibility that *B. seriolae* also induces changes in the expression of pro-inflammatory and other immune-related genes. The EST based gene-expression research and usage of EST-SNP polymorphism will help to determine the functional mechanisms as the key to a solution about parasite disease in yellowtail.

The ancestral chromosome and syntenic gene, or expression candidate gene information has been used to assess fine QTL mapping and to determine candidate gene loci for economically important traits in domestic animals and crops research. But the discovery of major QTL influencing susceptibility to *B. seriolae* represents a significant step forward for a better understanding of the host response to parasite infection, which in turn will assist fine QTL mapping and MAS studies for future yellowtail breeding programs.

MAS will greatly increase the efficiency and effectiveness of breeding compared to traditional breeding programs. The fundamental advantage of MAS compared to conventional phenotypic selection is that it is faster since the early selection could be made without collecting the phenotypic data, and fish will be selected with high reliability, and successive generations will enable avoidance of inbreeding depression.

In addition, the potential of MAS and marker-assisted introgression (MAI) use for disease risk management of marine aquaculture finfish can be considered. Wild aquatic species are not selected and still maintain high genetic diversities. Individuals have high potential for genetic breeding regarding phenotypic variation. Natural populations will be more appropriate to contribute to those genetic resources to find large QTL effects than strain populations, as breeding materials. Therefore, there is a real possibility that marker-assisted selection can be a success for aquatic species.

## Materials and Methods

### Ethics Statement

Field permits are not required for this species in Japan. The fish handling, husbandry and sampling methods were approved by Institutional Animal Care and Use Committee of National Research Institute of Aquaculture (IACUC-NRIA No. 03).

### Fish Families and Samples for QTL Analysis

Juvenile fish of *S. quinqueradiata* (100–120 mm in total length) were captured in the coastal waters of Goto Fukue island (Tsushima Strait, Nagasaki Prefecture). The yellowtail juveniles were purchased from commercial fishermen who catch wild juvenile yellowtail to supply fish farms for culture (artificial method of rearing from eggs for this species still does not allow a stable supply for practical use, and the aquaculture industry relies on wild caught juveniles for on-growing). One thousand juveniles were kept in a growing fish pen for two years. Two hundred of the three year old fish were pit tagged, and the number of *B. seriolae* counted six times to select the parents (data not shown). The selected fish were reared until maturity. We prepared two F_1_ families (family A and family B) for QTL analysis. Both families are pair-mating from different parents. To evaluate Benedenia disease resistance, artificial infections of *B. seriolae* were performed in about 100 progeny of each F_1_ family. For the genome-wide linkage analyses, 90 fishes of family A were used for finding candidate QTL, and 93 fishes of family B were used to confirm the QTL which are reproducible in the other family.

### Parasite Collection and Artificial Infection Experiment

*B. seriolae* used for artificial infection were collected from adult fish stock in growing pens. Mesh nets were hung in fish pens in which adult fish were parasitized by *B. seriolae*. Eggs of *B. seriolae* stuck to the net and a method to collect only *B. seriolae* was established and confirmed under experimental conditions several times for reproducibility [10]. Collected *B. seriolae* eggs could be induced to hatch by a 15 minute exposure to fluorescent light. Hatched larvae were kept in a shaded tank before infection. In preliminary experiments, it was confirmed that the parasite did not exist on their the test fishes bodies after they were soaked in a freshwater bath for five minutes. And the fish were allowed to recover from freshwater stress for two days before artificial infection. Hatched *B. seriolae* larvae were introduced to experimental fish tanks, and individual fish were exposed to two hundred larvae of *B. seriolae*. Water temperature was kept at 25.3–25.6°C during the infection experiment for 10 days until the larvae grew to the countable adult stage.

### Phenotypic Measurement of External Parasitism Resistance Traits Recorded

Ten days after exposure to *B. seriolae*, each fish was individually dipped in a tank of freshwater to remove the parasites. Thus, in the context of this study we recorded the total number of *B. seriolae* per fish. Benedenia disease frequency conformed to a normal phenotypic distribution as shown in Figure S3. Phenotypic information was used as quantitative trait values after linkage analysis (File S1). Total number of *B. seriolae* per fish was used as phenotypic information for QTL analysis. Number of pathogens for both families was normally distributed (Shapiro–Wilk test). Number of parasites was converted as Z score in each fish, in this case Z score was calculated as: number of parasites on each fish – average number of parasites/*standard deviation*.

### Phenotypic Measurement of Total Length, Body Length, Body Weight, and Surface Area Recorded

We measured the fish total length, body length, body weight, and surface area, which conformed to a normal phenotypic distribution as shown in Figure S4. The average length and weight across all F_1_ progeny were 185±5 mm and 65±10 g, and the surface area was calculated as (surface area) = 0.109×2×(total length)^2.113^ [31]. Furthemore we have to know the correlation of these factors with the number of *B. seriolae* parasites. The phenotypic information of fish size was used as quantitative trait values for genome-wide analysis (File S1).

### Data Collection and Genotyping

Microsatellite genotyping was performed in a 10 µl reaction volume containing 0.5 pmol/µl of unlabeled primer, 0.05 pmol/µl of fluorescence-end-labeled primer with [5′-TET], plus 1×buffer, 2.0 mM MgCl_2_, 0.2 mM dNTP, 1% BSA, 0.025 U of Taq DNA polymerase (Takara: Ex-Taq) and 25 ng template DNA. Suitable annealing temperatures for each microsatellite marker were used. PCR was performed on a MJ PTC-100 (Bio-Rad), and the program conditions were 95°C for 2 min for initial denaturation, followed by 35 cycles of 30 s at 95°C, 1 min at the annealing temperature (56–58°C), 1 min at 72°C and 3 min at 72°C for final extension. Amplification products were mixed with an equal volume of loading buffer [98% formamide, 10 mM EDTA (pH 8.0), 0.05% bromophenol blue], heated for 5 min at 95°C and then immediately cooled on ice. The mixture was loaded onto 6% PAGE-PLUS gel (Amresco, OH, USA) containing 7 M urea and 0.5×TBE buffer. Electrophoresis was performed in 0.5×TBE buffer at 1800 V constant voltage for 1.5 h. After electrophoresis, the gel was scanned and imaged using an FMBIO III Multi-View fluorescence image analyzer (Hitachi-soft, Tokyo, Japan).

Single Nucleotide Polymorphism (SNP) loci from new expressed sequence tag (EST) sequences of *S. quinqueradiata* (Kai *et al*., unpublished data) were used. To identify the polymorphism on SNP sites, we directly sequenced the PCR products of SNP regions in the parent of mapping family A using Sanger sequencer. The regions of polymorphic SNPs in the parents were also sequenced in 90 progeny of family A. Details of the microsatellite and SNP markers included in the linkage map are shown in File S1 and Figure S5.

A total of 1002 polymorphic markers in 24 linkage groups were used for family A using 860 microsatellite and 142 SNP markers. These marker locations and groups likely encompass all chromosomes for this cross. Nomenclature for the linkage groups was based on chromosome names using the linkage map [6]. Marker sex-specific map positions and genotypes for family A can be found in Figure S5 and File S1.

### Linkage and QTL Analyses

Genotype scoring was performed by using LINKMFEX ver. 2.3 application package [32]. The application can separate the allele genotypes which originate from males or females, and check the accuracy of genotypes in their progeny from parental male and female alleles to avoid genotype scoring errors. Linkage analysis was performed using genotype data converted to a backcross format. As grandparent genotypes were unknown, pairwise analyses were performed, and markers were sorted in linkage groups at LOD threshold of 5.0. Linkage phases were determined retrospectively by examining the assortment of alleles among linked markers. Then the allele was tested for goodness-of-fit for Mendelian segregation distortion using χ^2^-analysis. Also the order of the marker loci was confirmed to be correctly positioned, and was checked by double recombination events with the software application program in Map Manager QTX [33]. Graphic representations of linkage groups were generated with MAPCHART version 2.1 [34] using raw recombination fractions as estimates of map distances (Figure S5).

The estimated total genome-length of the female map is 1054.5 (cM) by Kosambi function, with an average of 34 markers per linkage group. In the male map, the estimated total genome length is 1054.0 (cM) by Kosambi function, with an average of 32 markers per linkage group in this analysis. The female checked marker average distance was 1.29 cM in total length, and the male average distance was 1.35 cM in total length. The present map would be useful in some molecular studies for genome-wide linkage analysis, because the average inter-marker distances of each map were calculated on the basis of one marker for one cluster. Thus, the female and male maps had 249 and 281 unique positions, with estimated average intervals of 4.2 cM and 3.7 cM in the female and male maps, respectively, and offer sufficient marker density for QTL studies.

Phase-corrected genotypes using linkage group marker orders were established using LINKMFEX and GENOVECT [32] prior to QTL analysis (File S1). QTL effects were tested for the segregating alleles from the sires and dams separately for each interval or markers [example: Squ(linkage group)F; F is dam allele in female linkage group. Squ(linkage group)M; M is sire allele in male linkage group].And the results of sires and dams are reported in tabular and figure form. Further QTL modeling was carried out by analysis of variance (ANOVA) using the fitqtl function in R/qtl [35], [36]. Putative QTL genotypes were added to the model using conditional genotype probabilities calculated at 1 cM intervals, and a genotyping error rate of 1%. Estimates of QTL effects and percentage of variance explained were obtained by comparing the full model to the sub-model without QTL. A similar approach was used for estimating covariate effects.

### Estimation of QTL Region of Benedenia Disease Resistance

QTL analysis was conducted using MapQTL 5 software [37]. In the first genome-wide screening to find candidate loci, we used the Kruskal–Wallis (K–W) test initially to determine the significance level of all marker loci associated with the disease resistance phenotype for the family A. Limited significant loci associated with the disease resistance in the family A, which were analyzed in the linkage groups from chromosome-wide assessment in family B, because the candidate QTL region was reproducible in other families.

Then, all QTL analyses were carried out using simple interval mapping which was also used to identify the location of significant LOD max position. Simple interval mapping models were fitted in each parent using the Haley & Knott regression method [38] with conditional genotype probabilities calculated at 1 cM intervals and constant genotype error rate 1%. Multiple QTL model mapping was then used to reduce the background genetic noise and the influence of other QTLs from other chromosomes [39], [40]. Although the results of both analyses are reported in tabular form for completeness, the results from the multiple QTL model analysis are used in the figures and discussion.

### Significance Thresholds and Confidence Intervals

Experiment-wide “genome-wide and chromosome-wide” significance thresholds were derived from permutation estimates by dividing the nominal p-value by the total number of chromosomes examined in the study [41]. Permutation tests were performed (1,000 replicates) to determine the threshold for LOD using a type I error rate of *P* = 0.05, *P* = 0.01 and *P* = 0.001. Significant QTLs and regions were graphically visualized using the software MapChart 2.1 and MapQTL 5. QTL confidence intervals were estimated by 1.8-LOD support interval with 95% confidence interval probability coverage [42].

### Estimation of Other QTL Regions Related to Total Length, Body Length, Body Weight, and Surface Area

QTL analyses for total length, body length, body weight, and surface area were also performed using K–W test and simple interval mapping, i.e. the same method of linkage analysis was performed to detect QTL region for Benedenia disease resistance.

### Conclusion

We have discovered the first genetic evidence that contributes to detailing the phenotypic resistance to Benedenia disease in yellowtail. Furthermore we identified two chromosomal regions containing QTL (*BDR-1, BDR-2*) that were associated with Benedenia disease resistance. Two putative QTL associations, of medium to large effect of with Benedenia disease resistance, were localized to linkage groups Squ2 and Squ20. These two loci were responsible for ranging from 32.9 to 35.5% of the total phenotypic variation. The important finding of this study was detected as a single peak of QTL (*BDR-1*) associated with Benedenia disease resistance within the proximal region of linkage group Squ2. The QTL peak (Sequ1295BAC) was located at position 30.3 cM, with a 95% confidence interval that the QTL region lies within 10 cM of the most proximal marker from Sequ0672TUF to Sequ1067TUF by simple interval mapping. The results of multiple QTL model mapping indicated QTL region within 5.5 cM interval. Furthermore the QTL (*BDR-2*) significant region would exist as multiple QTL on Squ20, because results of A and B families are detected as two peaks about the significant region in linkage group Squ20. Finding the QTL region strongly supports the potential for success of MAS for this disease. Moreover, we also identified the QTL on Squ7 associated with fish size (total length, body length, body weight, surface area) in yellowtail. The results from our current study suggested a negative correlation between growth traits and the number of parasites. The results will help resolve the mechanism of resistance to this important disease of yellowtail.

## Supporting Information

Figure S1 Localization of significant markers for Benedenia disease resistance in linkage group Squ20M with family B. Squ(linkage group)M; marker distance in male map. Map positions and LOD scores are based on a simple interval mapping QTL analysis using the software MapQTL 5. Horizontal lines across each plot indicate LOD significance threshold, *P_c_*; chomosome-wide significance threshold.
(PDF)

Figure S2 Significant markers for body weight simple interval mapping in linkage group Squ7F with family A. Squ(linkage group)F; marker distance in female map. Map positions and LOD scores are based on a simple interval mapping QTL analysis using the software MapQTL 5. Horizontal lines across each plot indicate LOD significance threshold, *P_g_*; genome-wide significance threshold.
(PDF)

Figure S3 Benedenia disease frequency conformed to a normal phenotypic distribution (Shapiro-Wilk test).
(PDF)

Figure S4 Fish total length, body length, body weight and surface area conformed normal to a phenotypic distribution (Shapiro-Wilk test).
(PDF)

Figure S5 Details of microsatellite and SNP markers included in the linkage map. The marker positions of linkage map are identified in male and female sex-specific location.
(PDF)

File S1 Genotype of microsatellite and SNP markers included in family A and B. And the phenotypic information of fish was used as quantitative trait values for genome-wide analysis.
(XLS)
