# Supplementary figures and images for: Quantitative Trait Loci (QTL) Associated with Resistance to a Monogenean Parasite (Benedenia seriolae) in Yellowtail (Seriola quinqueradiata) through Genome Wide Analysis
Source: PLoS One. 2013 Jun 4;8(6):e64987. doi: 10.1371/journal.pone.0064987 (PMC3672171; doi:10.1371/journal.pone.0064987)

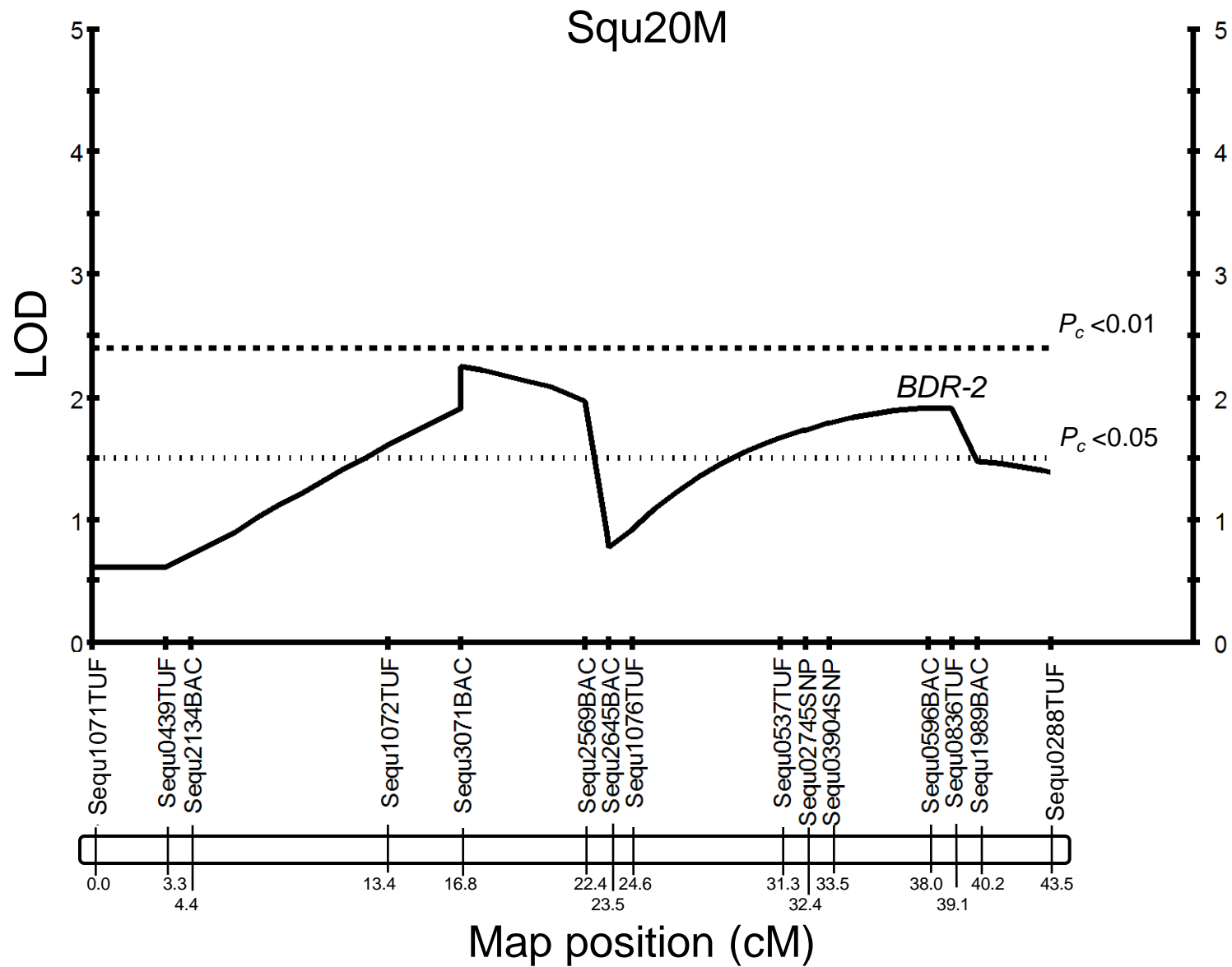

Figure S1

Supplement: Figure S1 — Localization of significant markers for Benedenia disease resistance in linkage group Squ20M with family B. Squ(linkage group)M; marker distance in male map. Map positions and LOD scores are based on a simple interval mapping QTL analysis using the software MapQTL 5. Horizontal lines across each plot indicate LOD significance threshold, Pc; chomosome-wide significance threshold. (PDF) [file pone.0064987.s001.pdf]

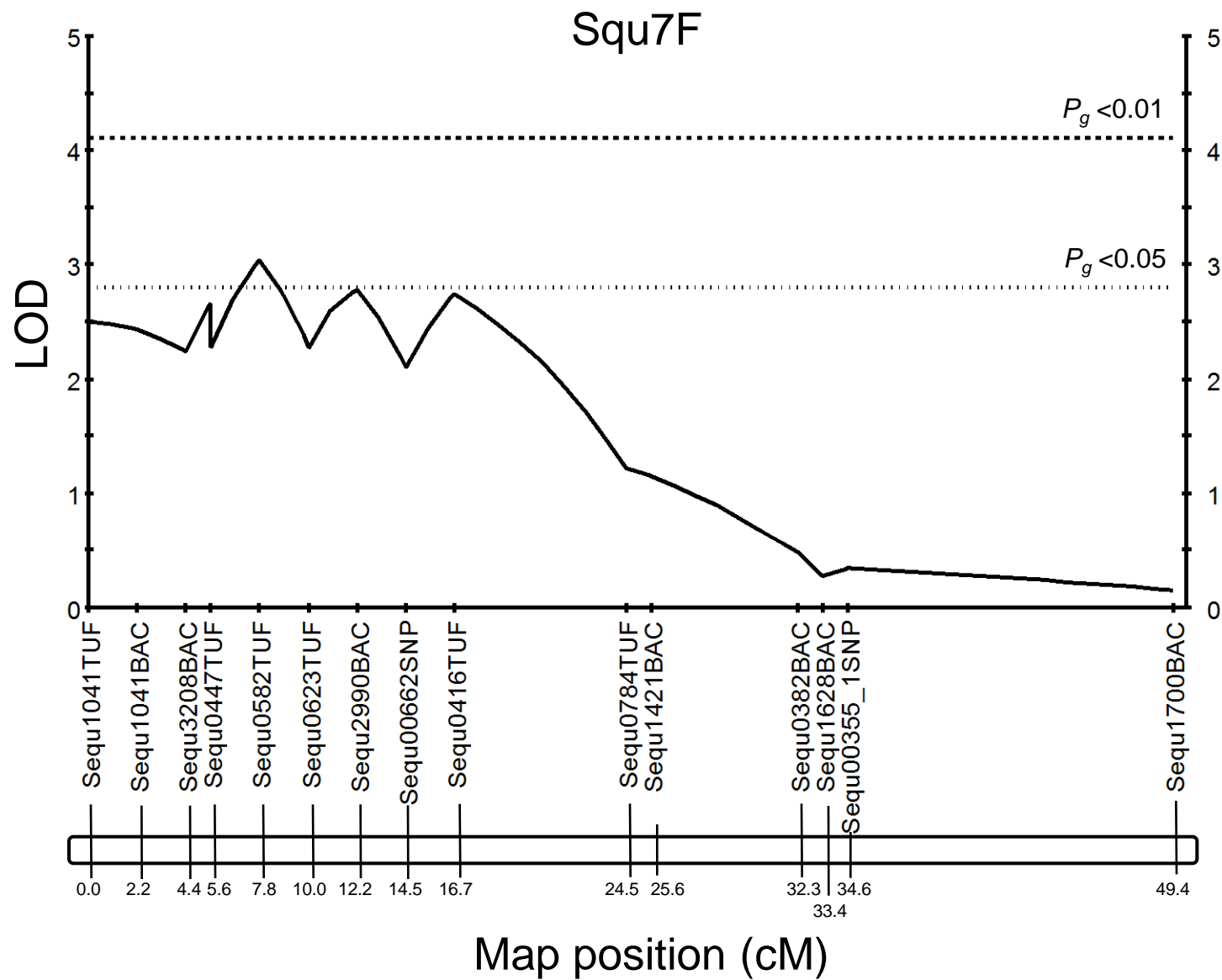

Figure S2

Supplement: Figure S2 — Significant markers for body weight simple interval mapping in linkage group Squ7F with family A. Squ(linkage group)F; marker distance in female map. Map positions and LOD scores are based on a simple interval mapping QTL analysis using the software MapQTL 5. Horizontal lines across each plot indicate LOD significance threshold, Pg; genome-wide significance threshold. (PDF) [file pone.0064987.s002.pdf]

## Family A

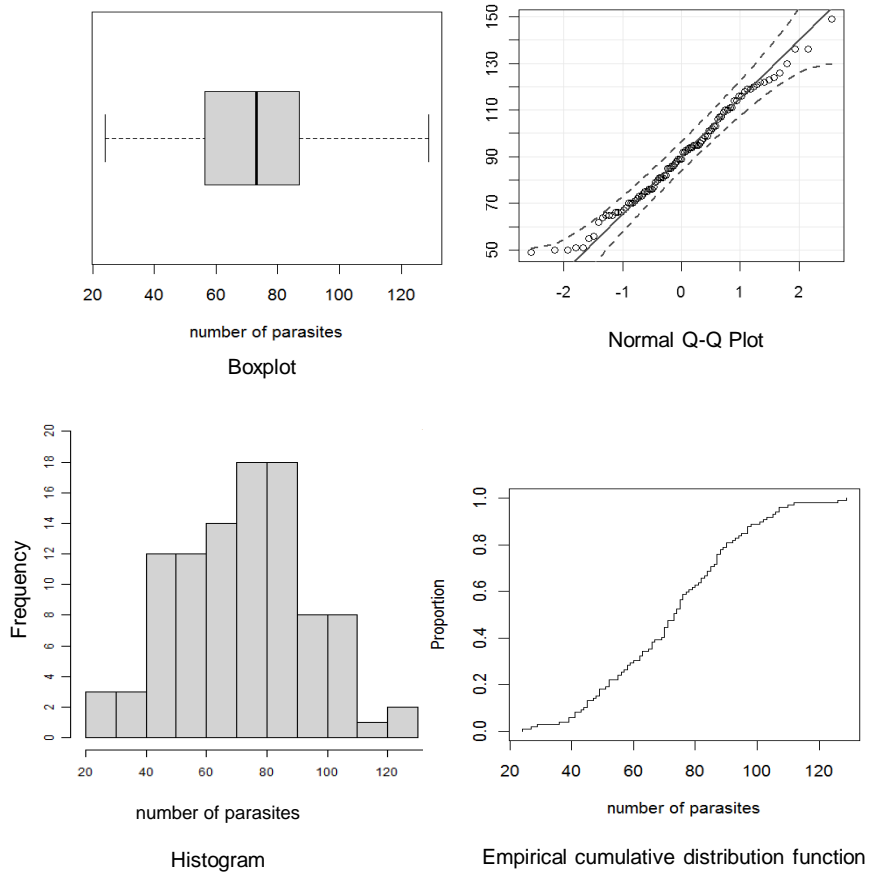

Shapiro-Wilk test:  $P$  value = 0.873

## Family B

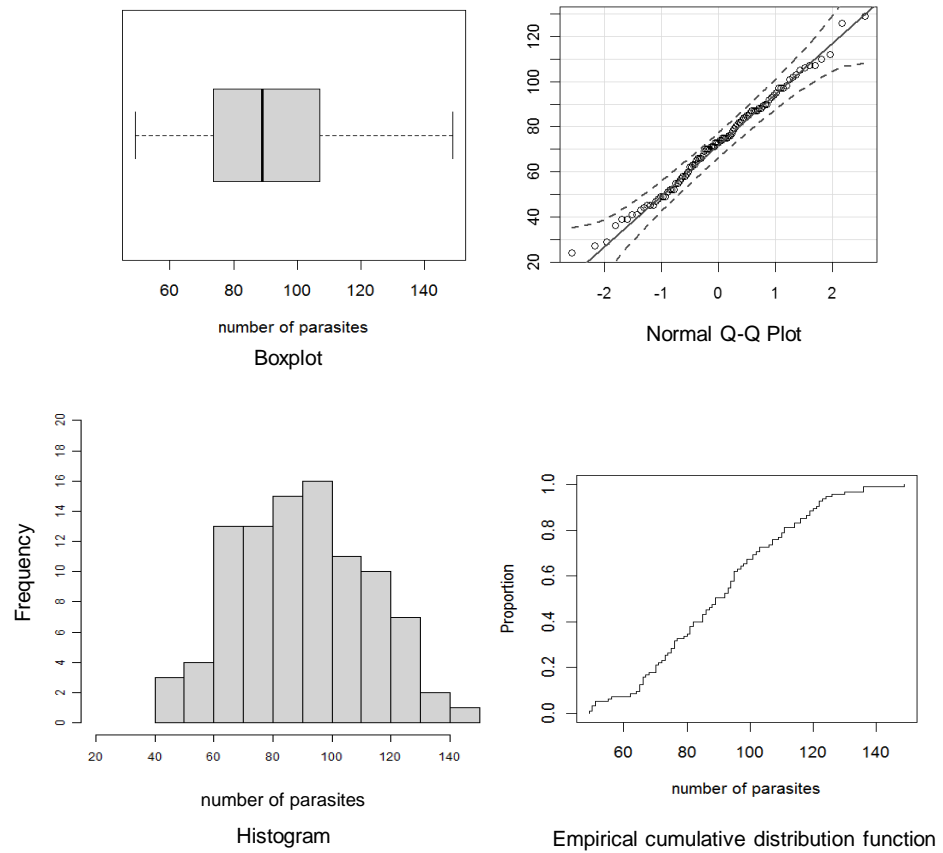

Shapiro-Wilk test:  $P$  value = 0.331

Figure S3

Supplement: Figure S3 — Benedenia disease frequency conformed to a normal phenotypic distribution (Shapiro-Wilk test). (PDF) [file pone.0064987.s003.pdf]

## Family A

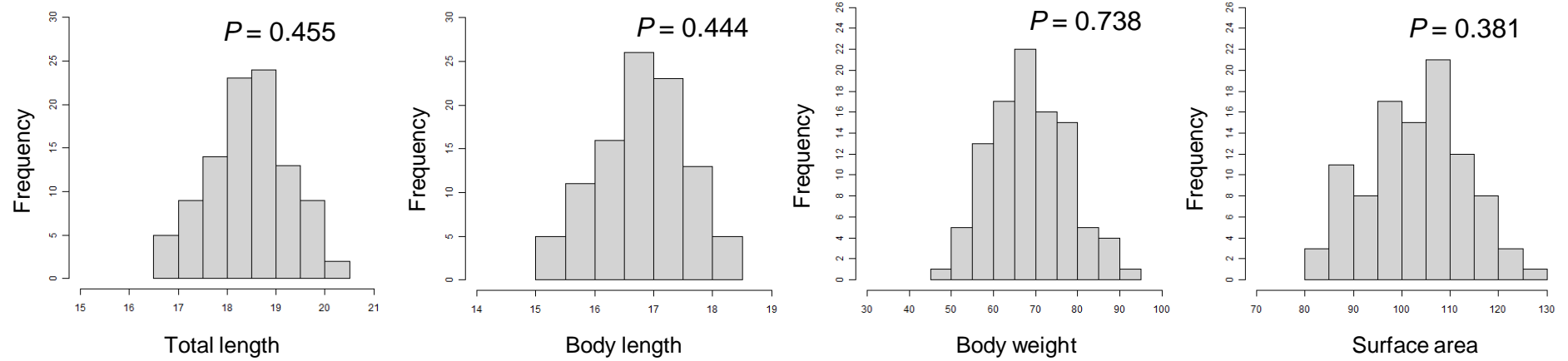

## Family B

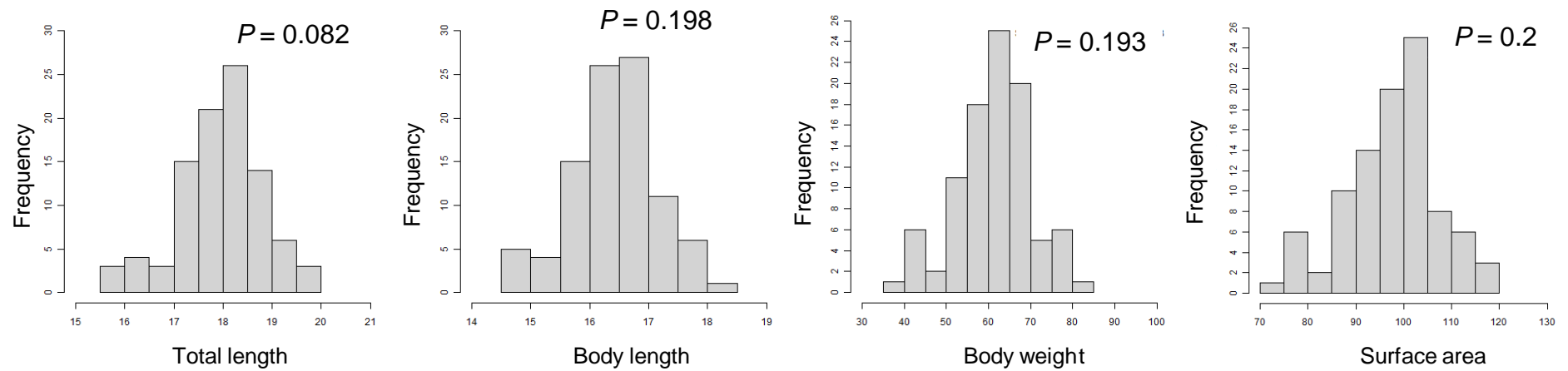

**Figure S4**

Supplement: Figure S4 — Fish total length, body length, body weight and surface area conformed normal to a phenotypic distribution (Shapiro-Wilk test). (PDF) [file pone.0064987.s004.pdf]
