# Supplementary material for: Quantitative Trait Loci (QTL) Associated with Resistance to a Monogenean Parasite (Benedenia seriolae) in Yellowtail (Seriola quinqueradiata) through Genome Wide Analysis
Source: PLoS One. 2013 Jun 4;8(6):e64987. doi: 10.1371/journal.pone.0064987 (PMC3672171; doi:10.1371/journal.pone.0064987)
Supplement: Figure S5 — Details of microsatellite and SNP markers included in the linkage map. The marker positions of linkage map are identified in male and female sex-specific location. (PDF) [file pone.0064987.s005.pdf]

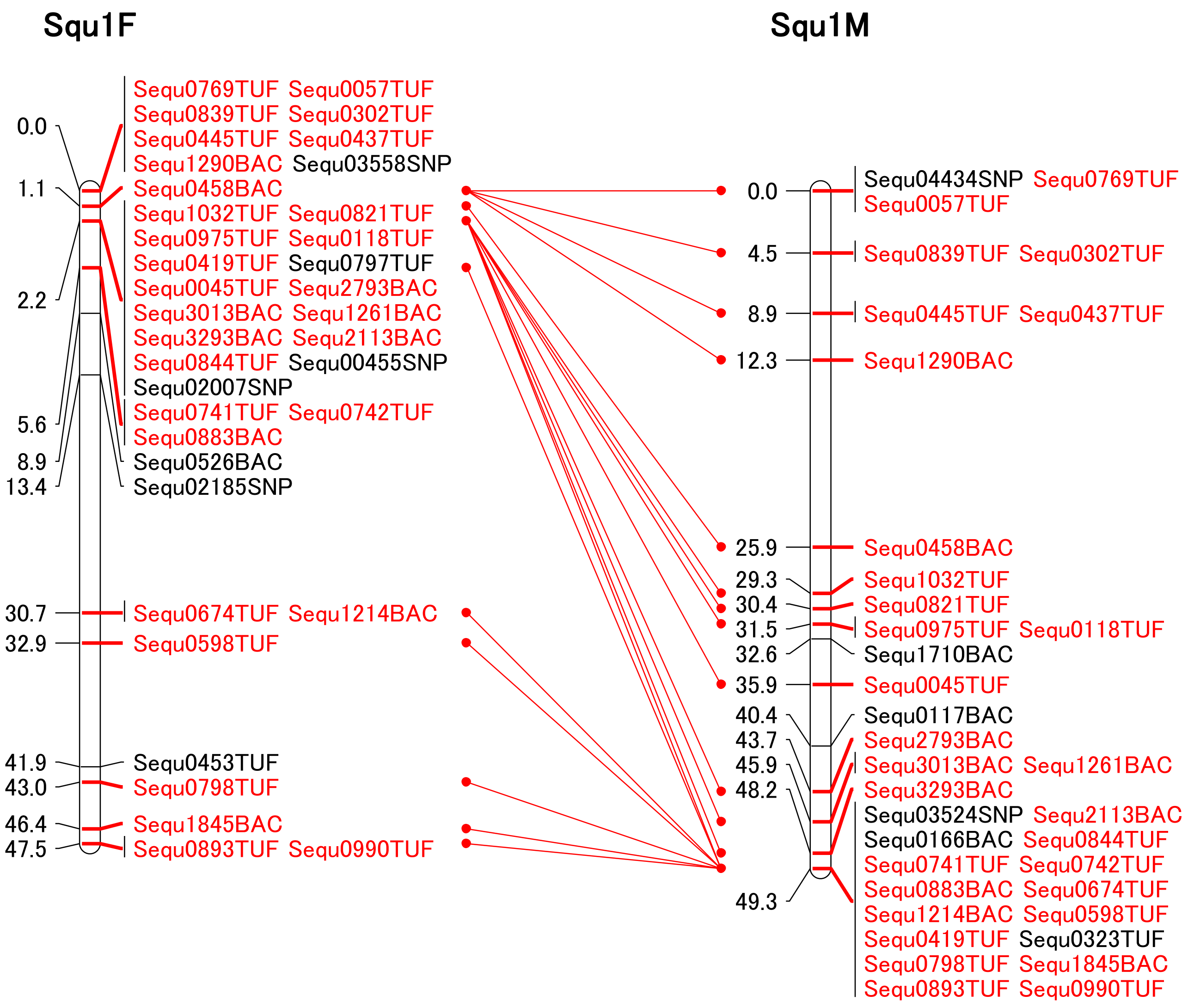
Squ1F
Squ1M
Sequ0769TUF Sequ0057TUF
Sequ0839TUF Sequ0302TUF
Sequ0445TUF Sequ0437TUF
Sequ1290BAC Sequ03558SNP
Sequ0458BAC
Sequ1032TUF Sequ0821TUF
Sequ0975TUF Sequ0118TUF
Sequ0419TUF Sequ0797TUF
Sequ0045TUF Sequ2793BAC
Sequ3013BAC Sequ1261BAC
Sequ3293BAC Sequ2113BAC
Sequ0844TUF Sequ00455SNP
Sequ02007SNP
Sequ0741TUF Sequ0742TUF
Sequ0883BAC
Sequ0526BAC
Sequ02185SNP
Sequ0674TUF Sequ1214BAC
Sequ0598TUF
Sequ0453TUF
Sequ0798TUF
Sequ1845BAC
Sequ0893TUF Sequ0990TUF
0.0
1.1
2.2
5.6
8.9
13.4
30.7
32.9
41.9
43.0
46.4
47.5
Sequ04434SNP Sequ0769TUF
Sequ0057TUF
Sequ0839TUF Sequ0302TUF
Sequ0445TUF Sequ0437TUF
Sequ1290BAC
Sequ0458BAC
Sequ1032TUF
Sequ0821TUF
Sequ0975TUF Sequ0118TUF
Sequ1710BAC
Sequ0045TUF
Sequ0117BAC
Sequ2793BAC
Sequ3013BAC Sequ1261BAC
Sequ3293BAC
Sequ03524SNP Sequ2113BAC
Sequ0166BAC Sequ0844TUF
Sequ0741TUF Sequ0742TUF
Sequ0883BAC Sequ0674TUF
Sequ1214BAC Sequ0598TUF
Sequ0419TUF Sequ0323TUF
Sequ0798TUF Sequ1845BAC
Sequ0893TUF Sequ0990TUF
0.0
4.5
8.9
12.3
25.9
29.3
30.4
31.5
32.6
35.9
40.4
43.7
45.9
48.2
49.3

Figure S5

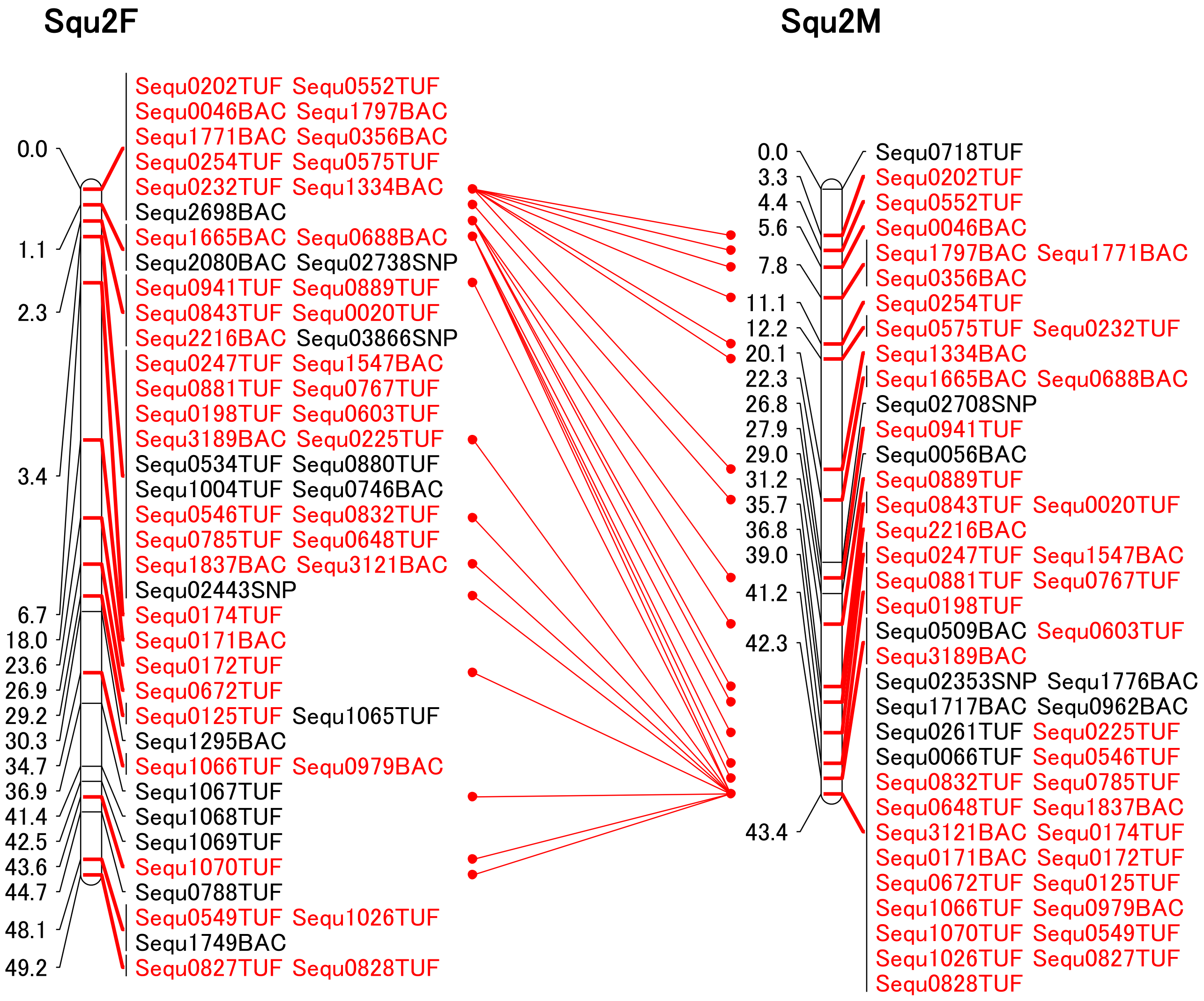

Squ2F
Squ2M
0.0
1.1
2.3
3.4
6.7
18.0
23.6
26.9
29.2
30.3
34.7
36.9
41.4
42.5
43.6
44.7
48.1
49.2
Sequ0202TUF Sequ0552TUF
Sequ0046BAC Sequ1797BAC
Sequ1771BAC Sequ0356BAC
Sequ0254TUF Sequ0575TUF
Sequ0232TUF Sequ1334BAC
Sequ2698BAC
Sequ1665BAC Sequ0688BAC
Sequ2080BAC Sequ02738SNP
Sequ0941TUF Sequ0889TUF
Sequ0843TUF Sequ0020TUF
Sequ2216BAC Sequ03866SNP
Sequ0247TUF Sequ1547BAC
Sequ0881TUF Sequ0767TUF
Sequ0198TUF Sequ0603TUF
Sequ3189BAC Sequ0225TUF
Sequ0534TUF Sequ0880TUF
Sequ1004TUF Sequ0746BAC
Sequ0546TUF Sequ0832TUF
Sequ0785TUF Sequ0648TUF
Sequ1837BAC Sequ3121BAC
Sequ02443SNP
Sequ0174TUF
Sequ0171BAC
Sequ0172TUF
Sequ0672TUF
Sequ0125TUF Sequ1065TUF
Sequ1295BAC
Sequ1066TUF Sequ0979BAC
Sequ1067TUF
Sequ1068TUF
Sequ1069TUF
Sequ1070TUF
Sequ0788TUF
Sequ0549TUF Sequ1026TUF
Sequ1749BAC
Sequ0827TUF Sequ0828TUF
0.0
3.3
4.4
5.6
7.8
11.1
12.2
20.1
22.3
26.8
27.9
29.0
31.2
35.7
36.8
39.0
41.2
42.3
43.4
Sequ0718TUF
Sequ0202TUF
Sequ0552TUF
Sequ0046BAC
Sequ1797BAC Sequ1771BAC
Sequ0356BAC
Sequ0254TUF
Sequ0575TUF Sequ0232TUF
Sequ1334BAC
Sequ1665BAC Sequ0688BAC
Sequ02708SNP
Sequ0941TUF
Sequ0056BAC
Sequ0889TUF
Sequ0843TUF Sequ0020TUF
Sequ2216BAC
Sequ0247TUF Sequ1547BAC
Sequ0881TUF Sequ0767TUF
Sequ0198TUF
Sequ0509BAC Sequ0603TUF
Sequ3189BAC
Sequ02353SNP Sequ1776BAC
Sequ1717BAC Sequ0962BAC
Sequ0261TUF Sequ0225TUF
Sequ0066TUF Sequ0546TUF
Sequ0832TUF Sequ0785TUF
Sequ0648TUF Sequ1837BAC
Sequ3121BAC Sequ0174TUF
Sequ0171BAC Sequ0172TUF
Sequ0672TUF Sequ0125TUF
Sequ1066TUF Sequ0979BAC
Sequ1070TUF Sequ0549TUF
Sequ1026TUF Sequ0827TUF
Sequ0828TUF

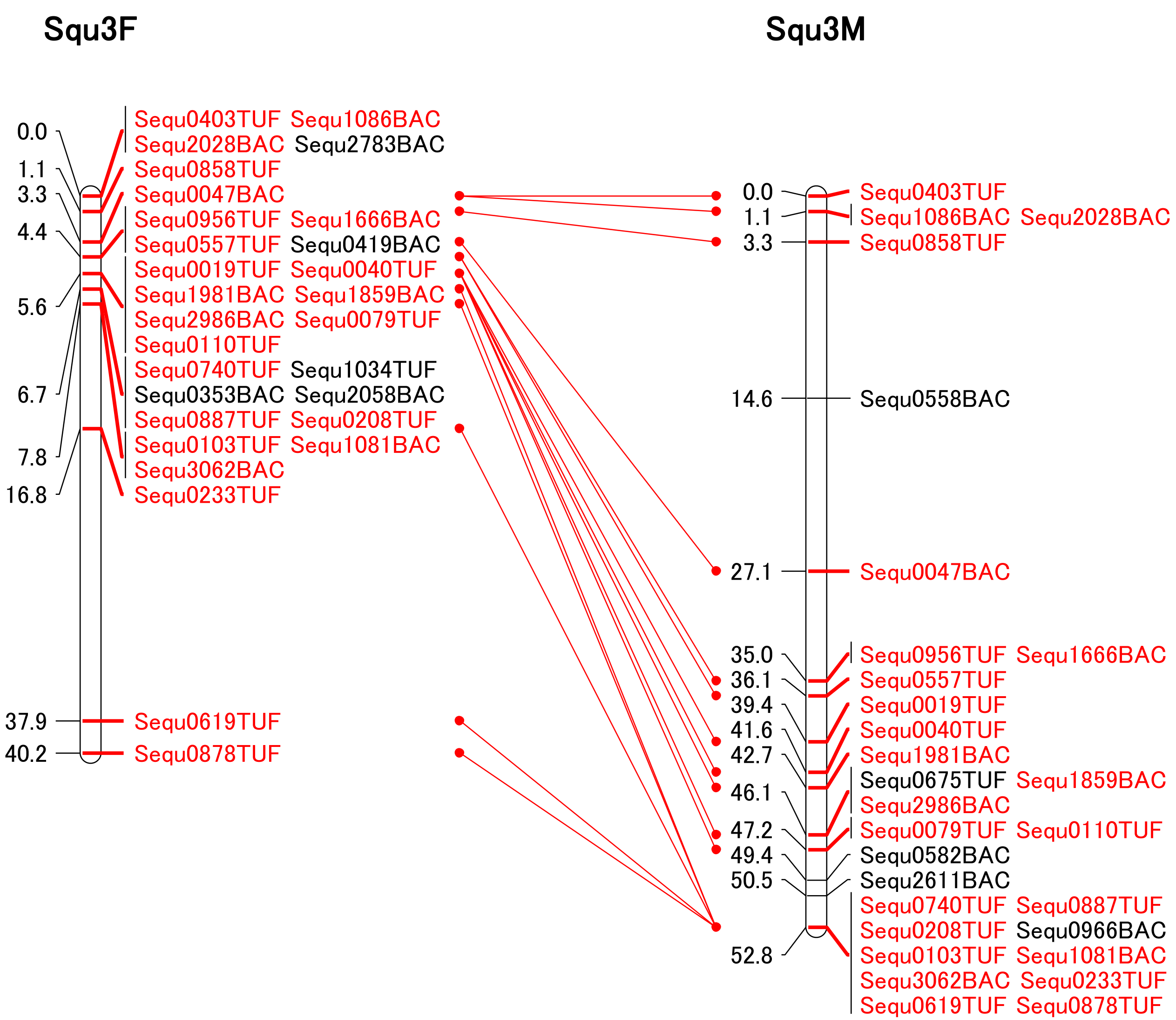
Squ3F
Squ3M
0.0
1.1
3.3
4.4
5.6
6.7
7.8
16.8
37.9
40.2
Sequ0403TUF Sequ1086BAC
Sequ2028BAC Sequ2783BAC
Sequ0858TUF
Sequ0047BAC
Sequ0956TUF Sequ1666BAC
Sequ0557TUF Sequ0419BAC
Sequ0019TUF Sequ0040TUF
Sequ1981BAC Sequ1859BAC
Sequ2986BAC Sequ0079TUF
Sequ0110TUF
Sequ0740TUF Sequ1034TUF
Sequ0353BAC Sequ2058BAC
Sequ0887TUF Sequ0208TUF
Sequ0103TUF Sequ1081BAC
Sequ3062BAC
Sequ0233TUF
Sequ0619TUF
Sequ0878TUF
0.0
1.1
3.3
14.6
27.1
35.0
36.1
39.4
41.6
42.7
46.1
47.2
49.4
50.5
52.8
Sequ0403TUF
Sequ1086BAC Sequ2028BAC
Sequ0858TUF
Sequ0558BAC
Sequ0047BAC
Sequ0956TUF Sequ1666BAC
Sequ0557TUF
Sequ0019TUF
Sequ0040TUF
Sequ1981BAC
Sequ0675TUF Sequ1859BAC
Sequ2986BAC
Sequ0079TUF Sequ0110TUF
Sequ0582BAC
Sequ2611BAC
Sequ0740TUF Sequ0887TUF
Sequ0208TUF Sequ0966BAC
Sequ0103TUF Sequ1081BAC
Sequ3062BAC Sequ0233TUF
Sequ0619TUF Sequ0878TUF

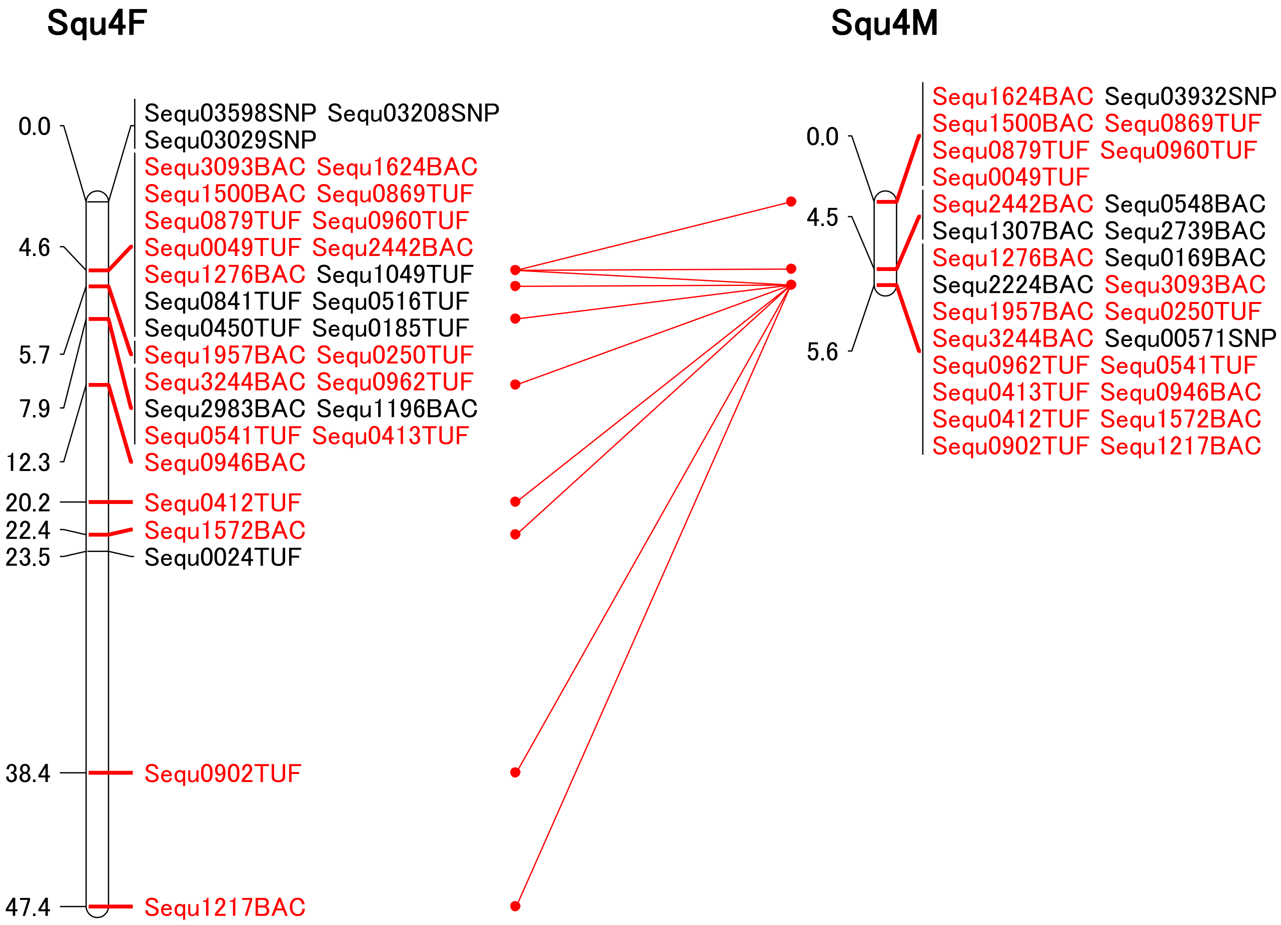

Squ4F
0.0
4.6
5.7
7.9
12.3
20.2
22.4
23.5
38.4
47.4
Sequ03598SNP Sequ03208SNP
Sequ03029SNP
Sequ3093BAC Sequ1624BAC
Sequ1500BAC Sequ0869TUF
Sequ0879TUF Sequ0960TUF
Sequ0049TUF Sequ2442BAC
Sequ1276BAC Sequ1049TUF
Sequ0841TUF Sequ0516TUF
Sequ0450TUF Sequ0185TUF
Sequ1957BAC Sequ0250TUF
Sequ3244BAC Sequ0962TUF
Sequ2983BAC Sequ1196BAC
Sequ0541TUF Sequ0413TUF
Sequ0946BAC
Sequ0412TUF
Sequ1572BAC
Sequ0024TUF
Sequ0902TUF
Sequ1217BAC
Squ4M
0.0
4.5
5.6
Sequ1624BAC Sequ03932SNP
Sequ1500BAC Sequ0869TUF
Sequ0879TUF Sequ0960TUF
Sequ0049TUF
Sequ2442BAC Sequ0548BAC
Sequ1307BAC Sequ2739BAC
Sequ1276BAC Sequ0169BAC
Sequ2224BAC Sequ3093BAC
Sequ1957BAC Sequ0250TUF
Sequ3244BAC Sequ00571SNP
Sequ0962TUF Sequ0541TUF
Sequ0413TUF Sequ0946BAC
Sequ0412TUF Sequ1572BAC
Sequ0902TUF Sequ1217BAC

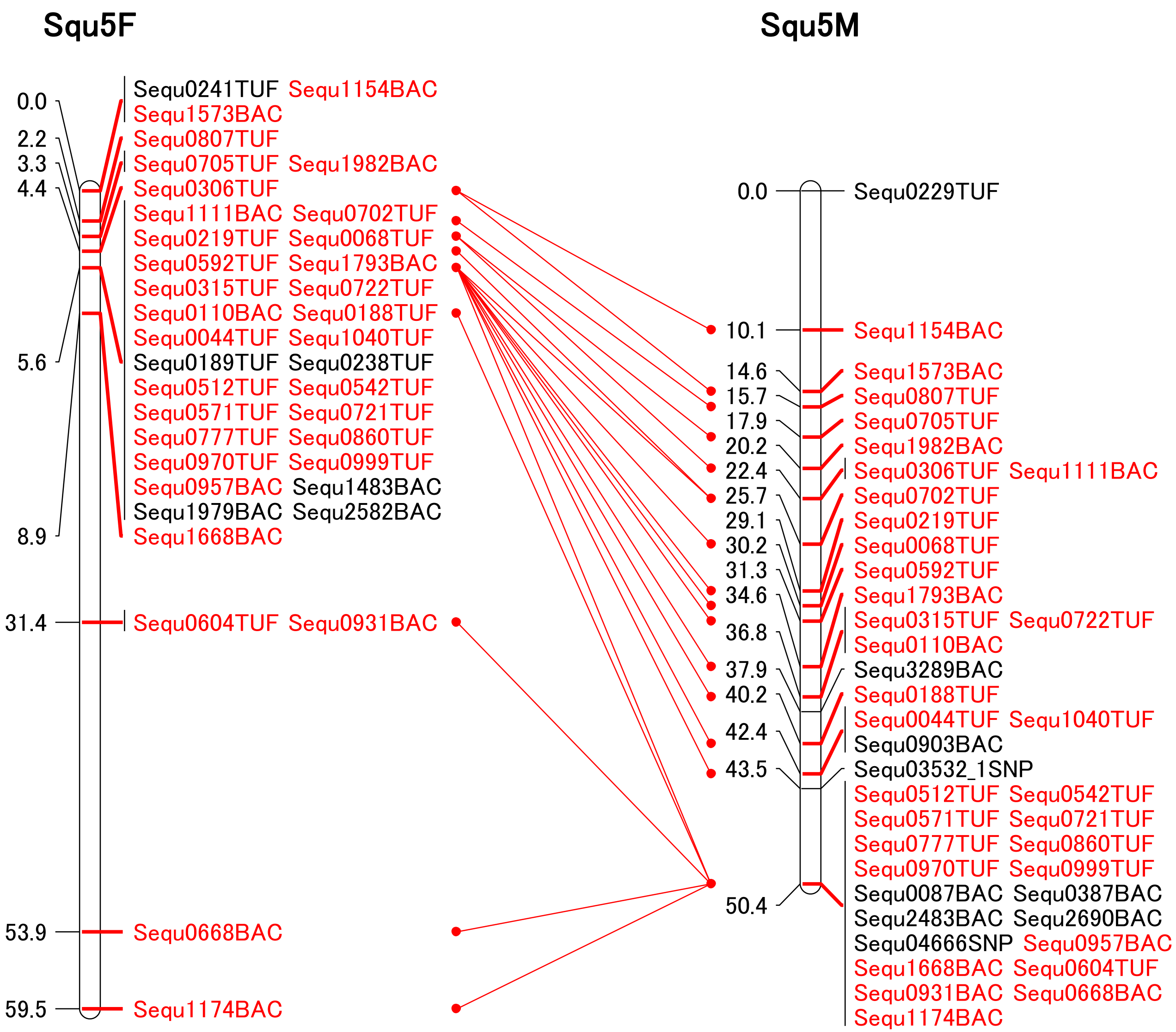

Squ5F
Squ5M
0.0 Sequ0241TUF Sequ1154BAC
Sequ1573BAC
2.2 Sequ0807TUF
3.3 Sequ0705TUF Sequ1982BAC
4.4 Sequ0306TUF
Sequ1111BAC Sequ0702TUF
Sequ0219TUF Sequ0068TUF
Sequ0592TUF Sequ1793BAC
Sequ0315TUF Sequ0722TUF
Sequ0110BAC Sequ0188TUF
Sequ0044TUF Sequ1040TUF
5.6 Sequ0189TUF Sequ0238TUF
Sequ0512TUF Sequ0542TUF
Sequ0571TUF Sequ0721TUF
Sequ0777TUF Sequ0860TUF
Sequ0970TUF Sequ0999TUF
Sequ0957BAC Sequ1483BAC
Sequ1979BAC Sequ2582BAC
8.9 Sequ1668BAC
31.4 Sequ0604TUF Sequ0931BAC
53.9 Sequ0668BAC
59.5 Sequ1174BAC
0.0 Sequ0229TUF
10.1 Sequ1154BAC
14.6 Sequ1573BAC
15.7 Sequ0807TUF
17.9 Sequ0705TUF
20.2 Sequ1982BAC
22.4 Sequ0306TUF Sequ1111BAC
25.7 Sequ0702TUF
29.1 Sequ0219TUF
30.2 Sequ0068TUF
31.3 Sequ0592TUF
34.6 Sequ1793BAC
Sequ0315TUF Sequ0722TUF
36.8 Sequ0110BAC
37.9 Sequ3289BAC
40.2 Sequ0188TUF
Sequ0044TUF Sequ1040TUF
42.4 Sequ0903BAC
43.5 Sequ03532_1SNP
Sequ0512TUF Sequ0542TUF
Sequ0571TUF Sequ0721TUF
Sequ0777TUF Sequ0860TUF
Sequ0970TUF Sequ0999TUF
50.4 Sequ0087BAC Sequ0387BAC
Sequ2483BAC Sequ2690BAC
Sequ04666SNP Sequ0957BAC
Sequ1668BAC Sequ0604TUF
Sequ0931BAC Sequ0668BAC
Sequ1174BAC

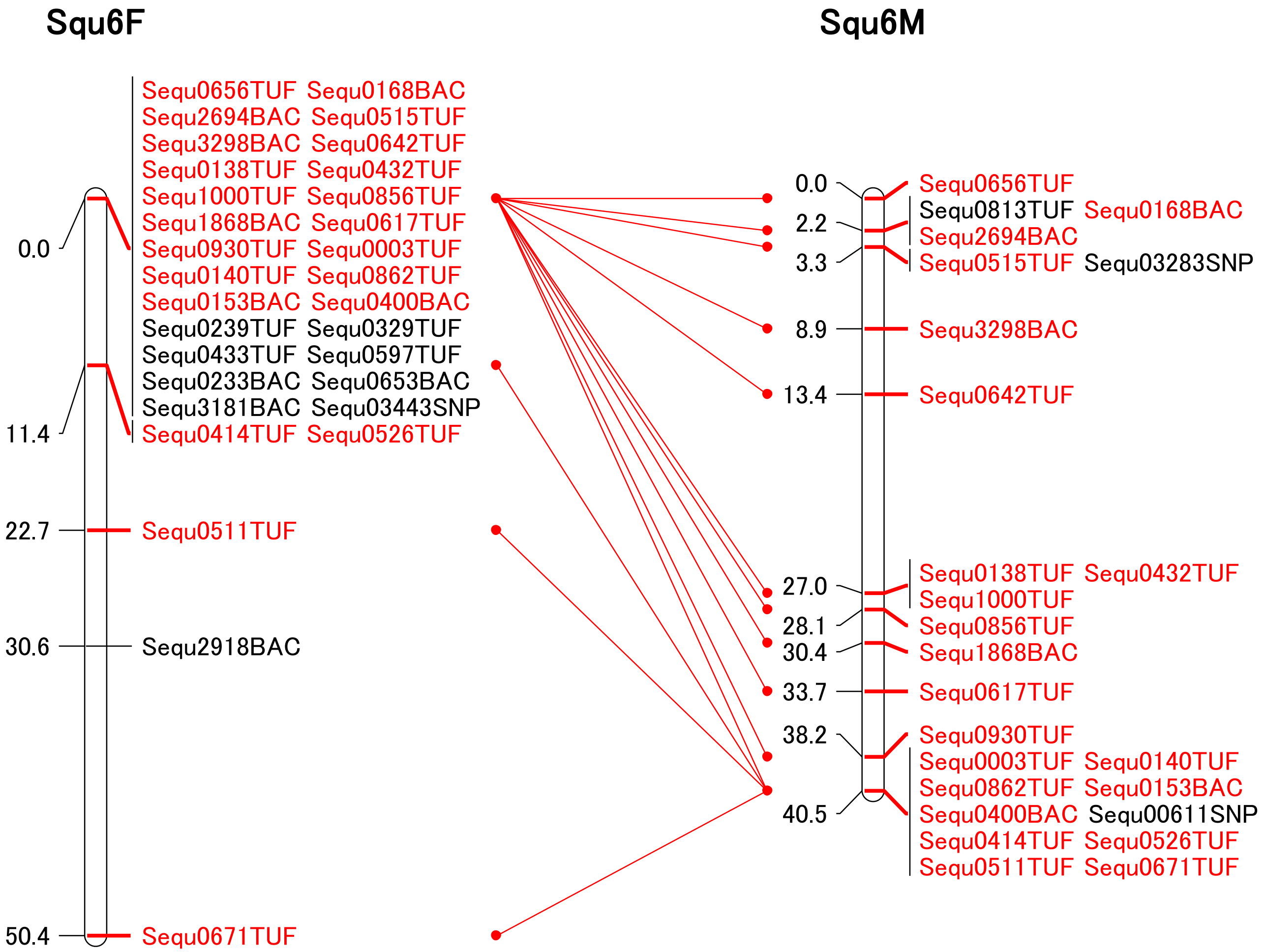
Squ6F
Squ6M
0.0
11.4
22.7
30.6
50.4
Sequ0656TUF Sequ0168BAC
Sequ2694BAC Sequ0515TUF
Sequ3298BAC Sequ0642TUF
Sequ0138TUF Sequ0432TUF
Sequ1000TUF Sequ0856TUF
Sequ1868BAC Sequ0617TUF
Sequ0930TUF Sequ0003TUF
Sequ0140TUF Sequ0862TUF
Sequ0153BAC Sequ0400BAC
Sequ0239TUF Sequ0329TUF
Sequ0433TUF Sequ0597TUF
Sequ0233BAC Sequ0653BAC
Sequ3181BAC Sequ03443SNP
Sequ0414TUF Sequ0526TUF
Sequ0511TUF
Sequ2918BAC
Sequ0671TUF
0.0
2.2
3.3
8.9
13.4
27.0
28.1
30.4
33.7
38.2
40.5
Sequ0656TUF
Sequ0813TUF Sequ0168BAC
Sequ2694BAC
Sequ0515TUF Sequ03283SNP
Sequ3298BAC
Sequ0642TUF
Sequ0138TUF Sequ0432TUF
Sequ1000TUF
Sequ0856TUF
Sequ1868BAC
Sequ0617TUF
Sequ0930TUF
Sequ0003TUF Sequ0140TUF
Sequ0862TUF Sequ0153BAC
Sequ0400BAC Sequ00611SNP
Sequ0414TUF Sequ0526TUF
Sequ0511TUF Sequ0671TUF

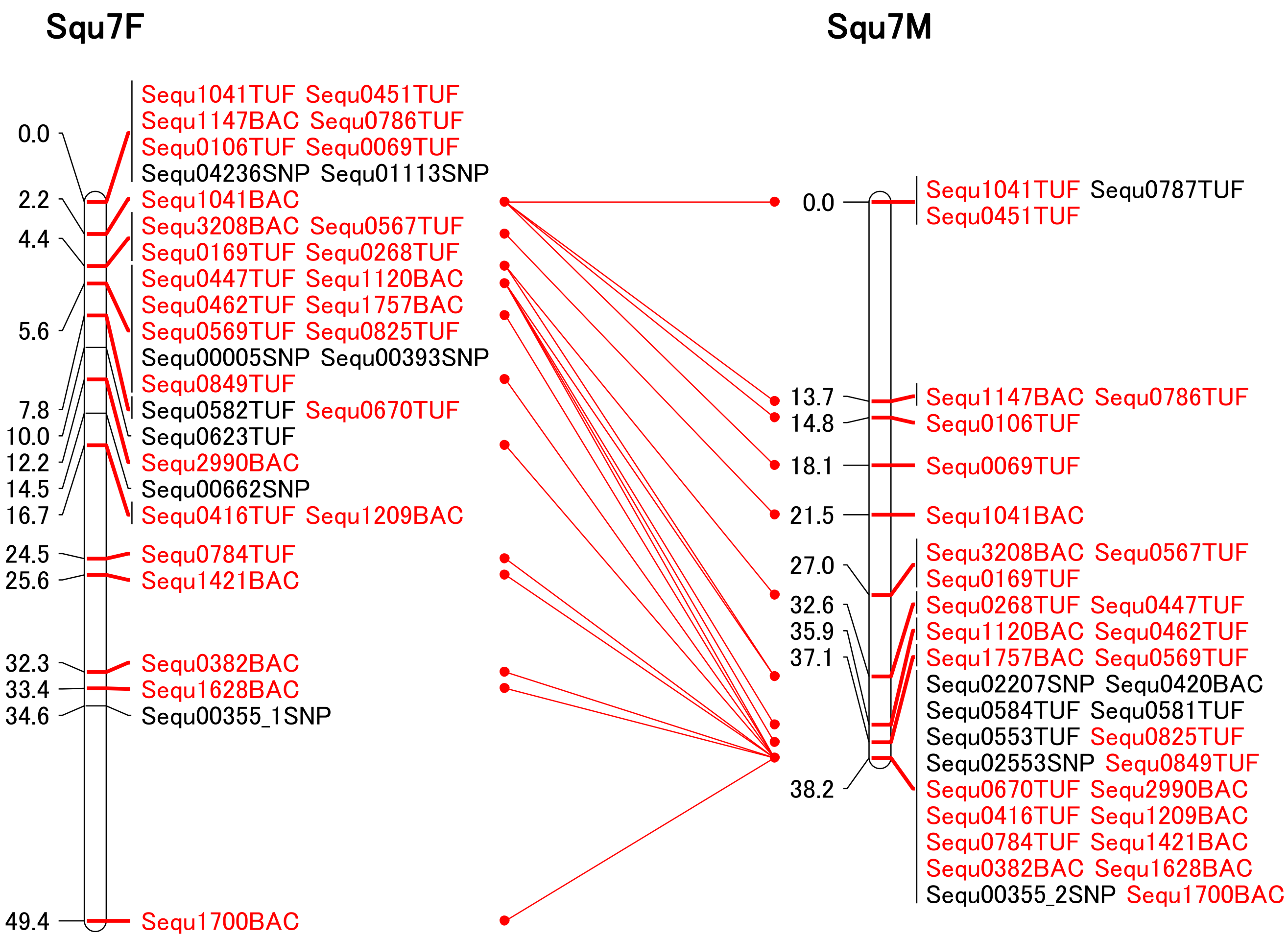
Squ7F
Squ7M
0.0
2.2
4.4
5.6
7.8
10.0
12.2
14.5
16.7
24.5
25.6
32.3
33.4
34.6
49.4
Sequ1041TUF Sequ0451TUF
Sequ1147BAC Sequ0786TUF
Sequ0106TUF Sequ0069TUF
Sequ04236SNP Sequ01113SNP
Sequ1041BAC
Sequ3208BAC Sequ0567TUF
Sequ0169TUF Sequ0268TUF
Sequ0447TUF Sequ1120BAC
Sequ0462TUF Sequ1757BAC
Sequ0569TUF Sequ0825TUF
Sequ00005SNP Sequ00393SNP
Sequ0849TUF
Sequ0582TUF Sequ0670TUF
Sequ0623TUF
Sequ2990BAC
Sequ00662SNP
Sequ0416TUF Sequ1209BAC
Sequ0784TUF
Sequ1421BAC
Sequ0382BAC
Sequ1628BAC
Sequ00355_1SNP
Sequ1700BAC
0.0
13.7
14.8
18.1
21.5
27.0
32.6
35.9
37.1
38.2
Sequ1041TUF Sequ0787TUF
Sequ0451TUF
Sequ1147BAC Sequ0786TUF
Sequ0106TUF
Sequ0069TUF
Sequ1041BAC
Sequ3208BAC Sequ0567TUF
Sequ0169TUF
Sequ0268TUF Sequ0447TUF
Sequ1120BAC Sequ0462TUF
Sequ1757BAC Sequ0569TUF
Sequ02207SNP Sequ0420BAC
Sequ0584TUF Sequ0581TUF
Sequ0553TUF Sequ0825TUF
Sequ02553SNP Sequ0849TUF
Sequ0670TUF Sequ2990BAC
Sequ0416TUF Sequ1209BAC
Sequ0784TUF Sequ1421BAC
Sequ0382BAC Sequ1628BAC
Sequ00355_2SNP Sequ1700BAC

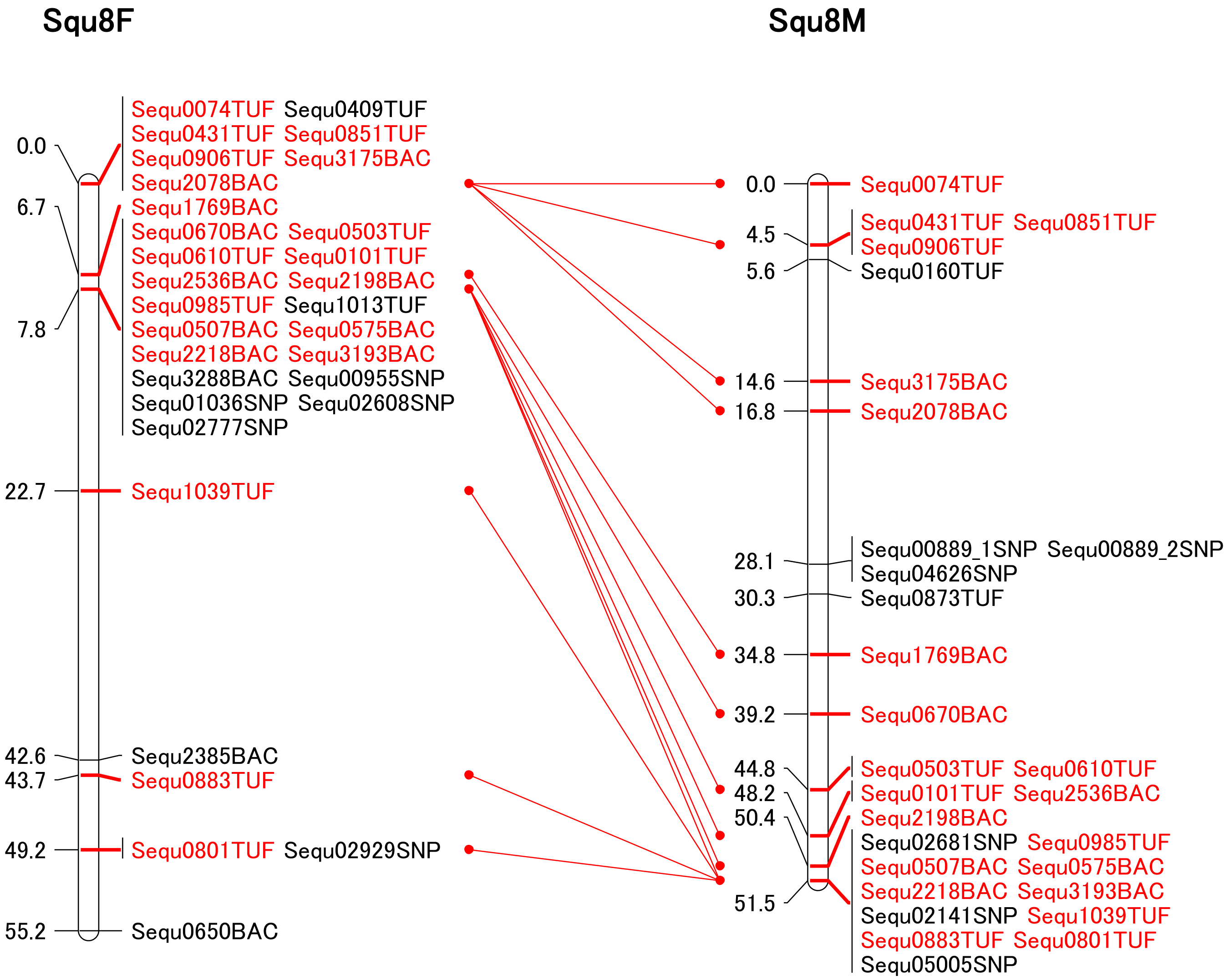

Squ8F
Squ8M
0.0
6.7
7.8
22.7
42.6
43.7
49.2
55.2
Sequ0074TUF Sequ0409TUF
Sequ0431TUF Sequ0851TUF
Sequ0906TUF Sequ3175BAC
Sequ2078BAC
Sequ1769BAC
Sequ0670BAC Sequ0503TUF
Sequ0610TUF Sequ0101TUF
Sequ2536BAC Sequ2198BAC
Sequ0985TUF Sequ1013TUF
Sequ0507BAC Sequ0575BAC
Sequ2218BAC Sequ3193BAC
Sequ3288BAC Sequ00955SNP
Sequ01036SNP Sequ02608SNP
Sequ02777SNP
Sequ1039TUF
Sequ2385BAC
Sequ0883TUF
Sequ0801TUF Sequ02929SNP
Sequ0650BAC
0.0
4.5
5.6
14.6
16.8
28.1
30.3
34.8
39.2
44.8
48.2
50.4
51.5
Sequ0074TUF
Sequ0431TUF Sequ0851TUF
Sequ0906TUF
Sequ0160TUF
Sequ3175BAC
Sequ2078BAC
Sequ00889_1SNP Sequ00889_2SNP
Sequ04626SNP
Sequ0873TUF
Sequ1769BAC
Sequ0670BAC
Sequ0503TUF Sequ0610TUF
Sequ0101TUF Sequ2536BAC
Sequ2198BAC
Sequ02681SNP Sequ0985TUF
Sequ0507BAC Sequ0575BAC
Sequ2218BAC Sequ3193BAC
Sequ02141SNP Sequ1039TUF
Sequ0883TUF Sequ0801TUF
Sequ05005SNP

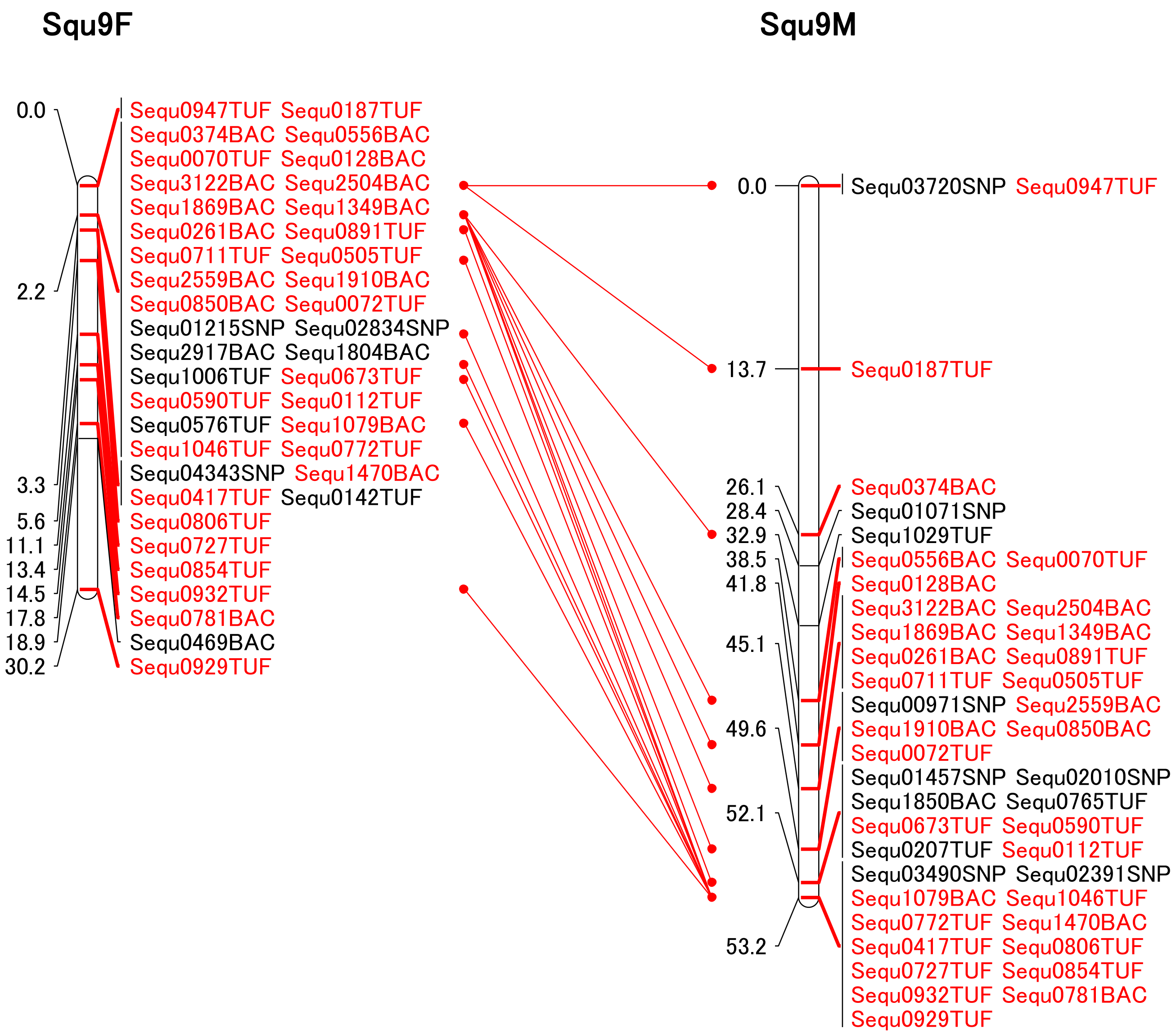

Squ9F
Squ9M
0.0
2.2
3.3
5.6
11.1
13.4
14.5
17.8
18.9
30.2
Sequ0947TUF Sequ0187TUF
Sequ0374BAC Sequ0556BAC
Sequ0070TUF Sequ0128BAC
Sequ3122BAC Sequ2504BAC
Sequ1869BAC Sequ1349BAC
Sequ0261BAC Sequ0891TUF
Sequ0711TUF Sequ0505TUF
Sequ2559BAC Sequ1910BAC
Sequ0850BAC Sequ0072TUF
Sequ01215SNP Sequ02834SNP
Sequ2917BAC Sequ1804BAC
Sequ1006TUF Sequ0673TUF
Sequ0590TUF Sequ0112TUF
Sequ0576TUF Sequ1079BAC
Sequ1046TUF Sequ0772TUF
Sequ04343SNP Sequ1470BAC
Sequ0417TUF Sequ0142TUF
Sequ0806TUF
Sequ0727TUF
Sequ0854TUF
Sequ0932TUF
Sequ0781BAC
Sequ0469BAC
Sequ0929TUF
0.0
13.7
26.1
28.4
32.9
38.5
41.8
45.1
49.6
52.1
53.2
Sequ03720SNP Sequ0947TUF
Sequ0187TUF
Sequ0374BAC
Sequ01071SNP
Sequ1029TUF
Sequ0556BAC Sequ0070TUF
Sequ0128BAC
Sequ3122BAC Sequ2504BAC
Sequ1869BAC Sequ1349BAC
Sequ0261BAC Sequ0891TUF
Sequ0711TUF Sequ0505TUF
Sequ00971SNP Sequ2559BAC
Sequ1910BAC Sequ0850BAC
Sequ0072TUF
Sequ01457SNP Sequ02010SNP
Sequ1850BAC Sequ0765TUF
Sequ0673TUF Sequ0590TUF
Sequ0207TUF Sequ0112TUF
Sequ03490SNP Sequ02391SNP
Sequ1079BAC Sequ1046TUF
Sequ0772TUF Sequ1470BAC
Sequ0417TUF Sequ0806TUF
Sequ0727TUF Sequ0854TUF
Sequ0932TUF Sequ0781BAC
Sequ0929TUF

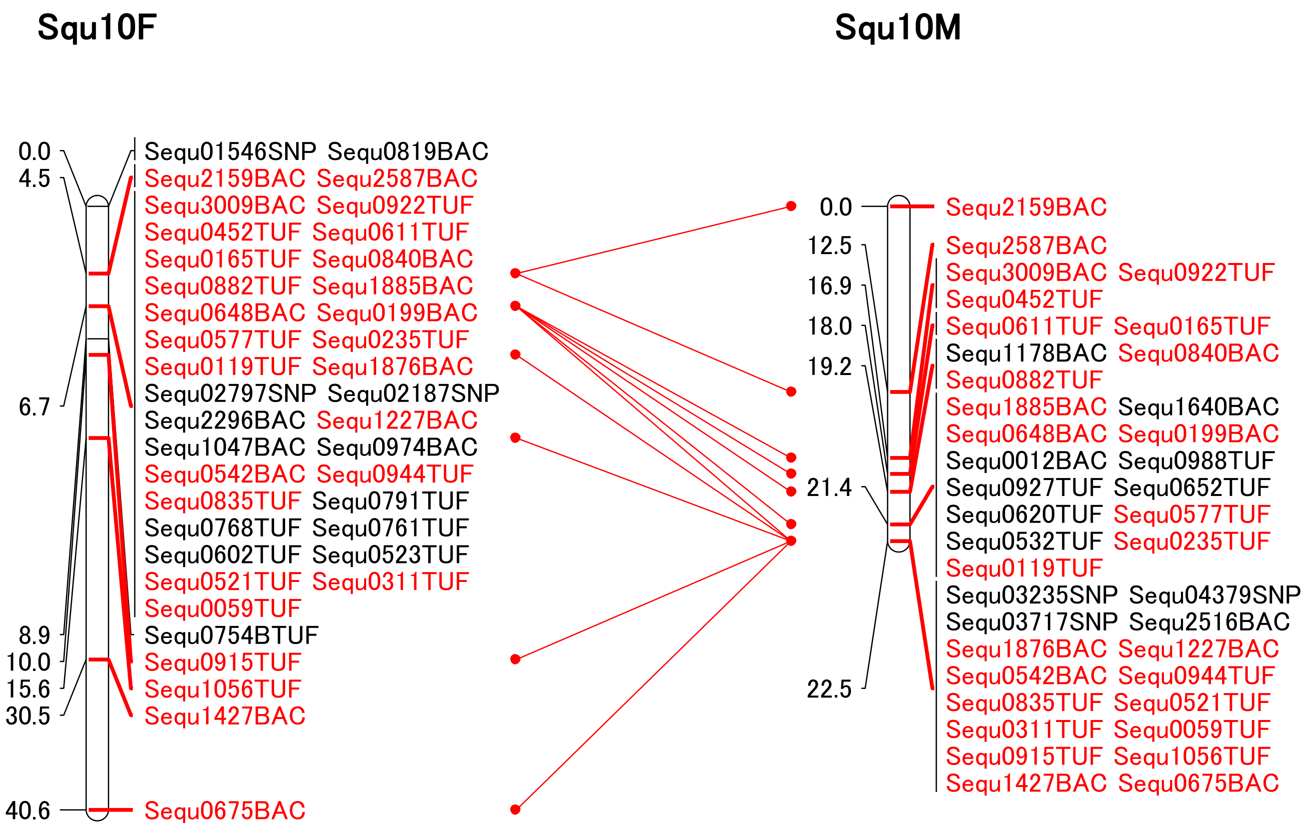

Squ10F
Squ10M
0.0 Sequ01546SNP Sequ0819BAC
4.5 Sequ2159BAC Sequ2587BAC
Sequ3009BAC Sequ0922TUF
Sequ0452TUF Sequ0611TUF
Sequ0165TUF Sequ0840BAC
Sequ0882TUF Sequ1885BAC
Sequ0648BAC Sequ0199BAC
Sequ0577TUF Sequ0235TUF
Sequ0119TUF Sequ1876BAC
6.7 Sequ02797SNP Sequ02187SNP
Sequ2296BAC Sequ1227BAC
Sequ1047BAC Sequ0974BAC
Sequ0542BAC Sequ0944TUF
Sequ0835TUF Sequ0791TUF
Sequ0768TUF Sequ0761TUF
Sequ0602TUF Sequ0523TUF
Sequ0521TUF Sequ0311TUF
Sequ0059TUF
8.9 Sequ0754BTUF
10.0 Sequ0915TUF
15.6 Sequ1056TUF
30.5 Sequ1427BAC
40.6 Sequ0675BAC
0.0 Sequ2159BAC
12.5 Sequ2587BAC
16.9 Sequ3009BAC Sequ0922TUF
18.0 Sequ0452TUF
19.2 Sequ0611TUF Sequ0165TUF
Sequ1178BAC Sequ0840BAC
Sequ0882TUF
Sequ1885BAC Sequ1640BAC
Sequ0648BAC Sequ0199BAC
Sequ0012BAC Sequ0988TUF
21.4 Sequ0927TUF Sequ0652TUF
Sequ0620TUF Sequ0577TUF
Sequ0532TUF Sequ0235TUF
Sequ0119TUF
Sequ03235SNP Sequ04379SNP
Sequ03717SNP Sequ2516BAC
Sequ1876BAC Sequ1227BAC
22.5 Sequ0542BAC Sequ0944TUF
Sequ0835TUF Sequ0521TUF
Sequ0311TUF Sequ0059TUF
Sequ0915TUF Sequ1056TUF
Sequ1427BAC Sequ0675BAC

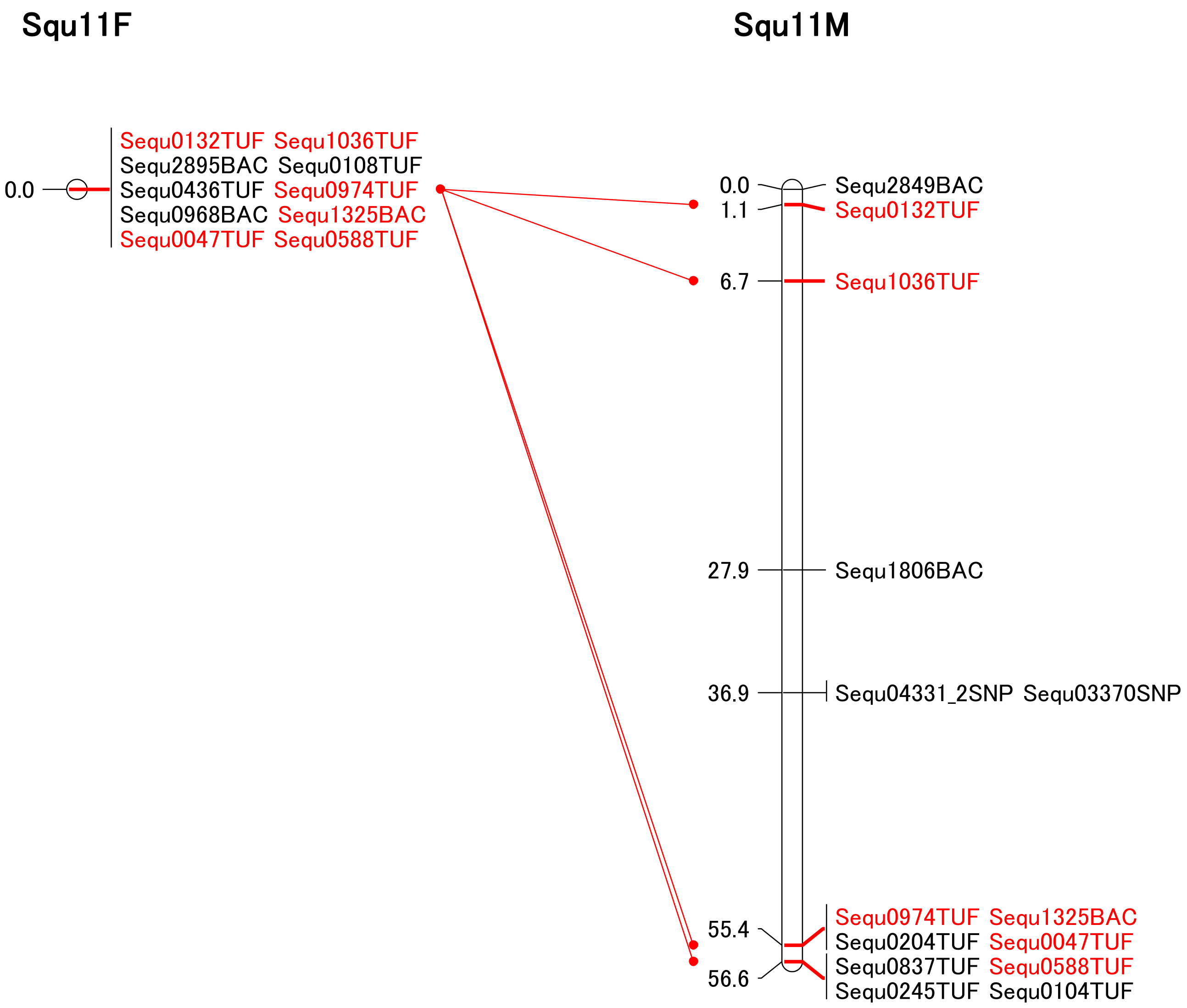
Squ11F
Squ11M
0.0
Sequ0132TUF Sequ1036TUF
Sequ2895BAC Sequ0108TUF
Sequ0436TUF Sequ0974TUF
Sequ0968BAC Sequ1325BAC
Sequ0047TUF Sequ0588TUF
0.0 Sequ2849BAC
1.1 Sequ0132TUF
6.7 Sequ1036TUF
27.9 Sequ1806BAC
36.9 Sequ04331_2SNP Sequ03370SNP
55.4
56.6
Sequ0974TUF Sequ1325BAC
Sequ0204TUF Sequ0047TUF
Sequ0837TUF Sequ0588TUF
Sequ0245TUF Sequ0104TUF

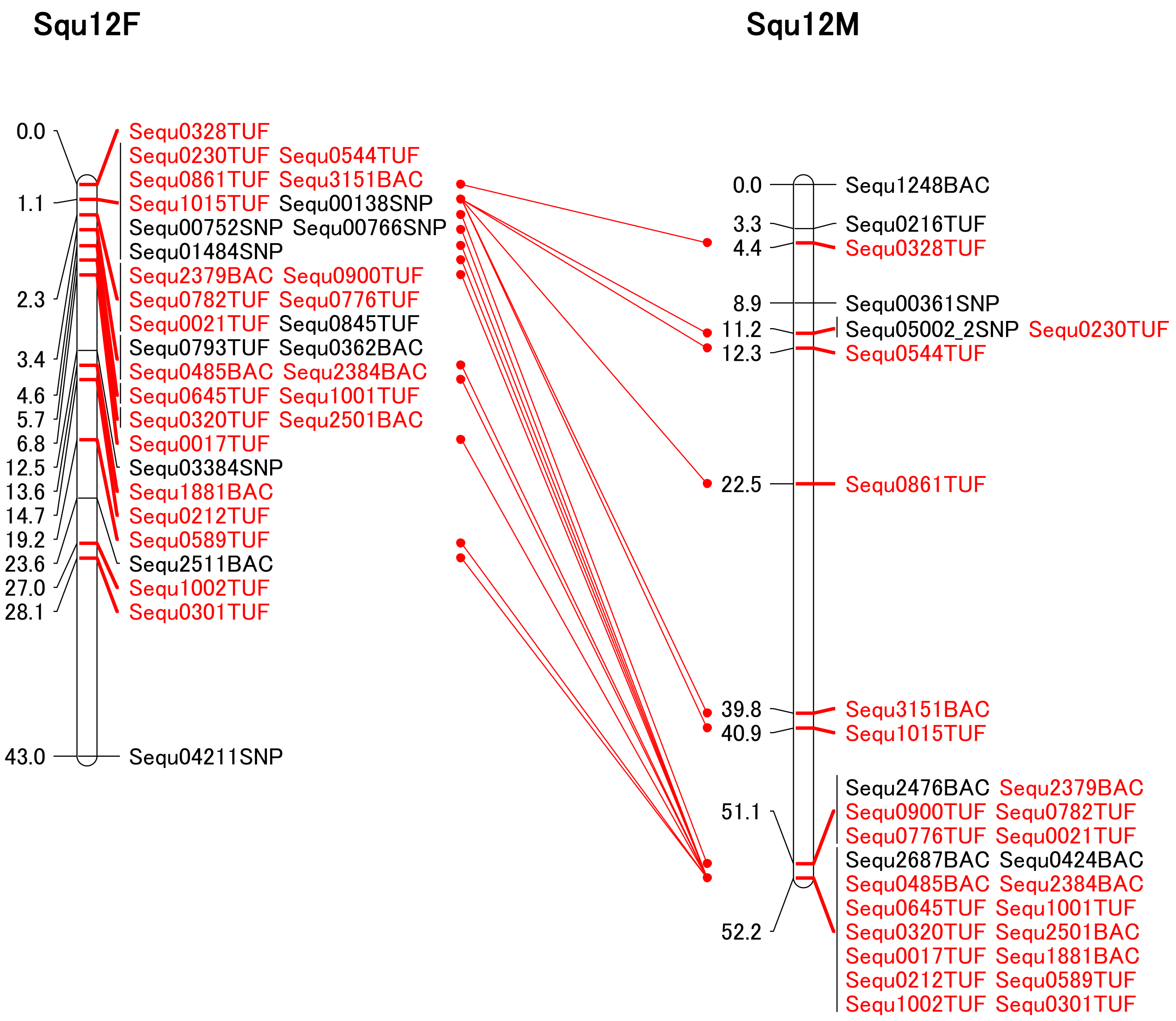

Squ12F
Squ12M
0.0
1.1
2.3
3.4
4.6
5.7
6.8
12.5
13.6
14.7
19.2
23.6
27.0
28.1
43.0
Sequ0328TUF
Sequ0230TUF Sequ0544TUF
Sequ0861TUF Sequ3151BAC
Sequ1015TUF Sequ00138SNP
Sequ00752SNP Sequ00766SNP
Sequ01484SNP
Sequ2379BAC Sequ0900TUF
Sequ0782TUF Sequ0776TUF
Sequ0021TUF Sequ0845TUF
Sequ0793TUF Sequ0362BAC
Sequ0485BAC Sequ2384BAC
Sequ0645TUF Sequ1001TUF
Sequ0320TUF Sequ2501BAC
Sequ0017TUF
Sequ03384SNP
Sequ1881BAC
Sequ0212TUF
Sequ0589TUF
Sequ2511BAC
Sequ1002TUF
Sequ0301TUF
Sequ04211SNP
0.0
3.3
4.4
8.9
11.2
12.3
22.5
39.8
40.9
51.1
52.2
Sequ1248BAC
Sequ0216TUF
Sequ0328TUF
Sequ00361SNP
Sequ05002_2SNP Sequ0230TUF
Sequ0544TUF
Sequ0861TUF
Sequ3151BAC
Sequ1015TUF
Sequ2476BAC Sequ2379BAC
Sequ0900TUF Sequ0782TUF
Sequ0776TUF Sequ0021TUF
Sequ2687BAC Sequ0424BAC
Sequ0485BAC Sequ2384BAC
Sequ0645TUF Sequ1001TUF
Sequ0320TUF Sequ2501BAC
Sequ0017TUF Sequ1881BAC
Sequ0212TUF Sequ0589TUF
Sequ1002TUF Sequ0301TUF

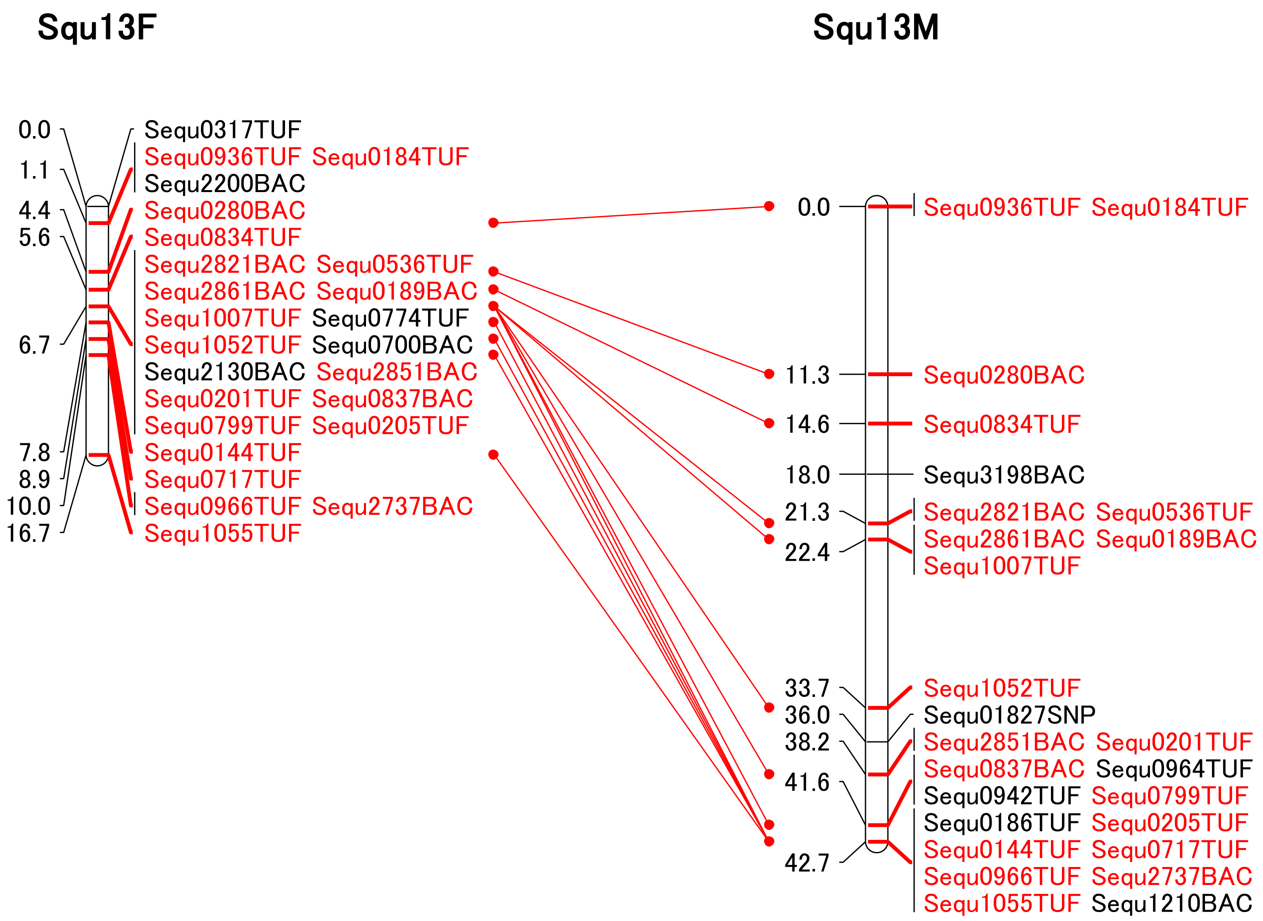

Squ13F
Squ13M
0.0
1.1
4.4
5.6
6.7
7.8
8.9
10.0
16.7
Sequ0317TUF
Sequ0936TUF Sequ0184TUF
Sequ2200BAC
Sequ0280BAC
Sequ0834TUF
Sequ2821BAC Sequ0536TUF
Sequ2861BAC Sequ0189BAC
Sequ1007TUF Sequ0774TUF
Sequ1052TUF Sequ0700BAC
Sequ2130BAC Sequ2851BAC
Sequ0201TUF Sequ0837BAC
Sequ0799TUF Sequ0205TUF
Sequ0144TUF
Sequ0717TUF
Sequ0966TUF Sequ2737BAC
Sequ1055TUF
0.0
11.3
14.6
18.0
21.3
22.4
33.7
36.0
38.2
41.6
42.7
Sequ0936TUF Sequ0184TUF
Sequ0280BAC
Sequ0834TUF
Sequ3198BAC
Sequ2821BAC Sequ0536TUF
Sequ2861BAC Sequ0189BAC
Sequ1007TUF
Sequ1052TUF
Sequ01827SNP
Sequ2851BAC Sequ0201TUF
Sequ0837BAC Sequ0964TUF
Sequ0942TUF Sequ0799TUF
Sequ0186TUF Sequ0205TUF
Sequ0144TUF Sequ0717TUF
Sequ0966TUF Sequ2737BAC
Sequ1055TUF Sequ1210BAC

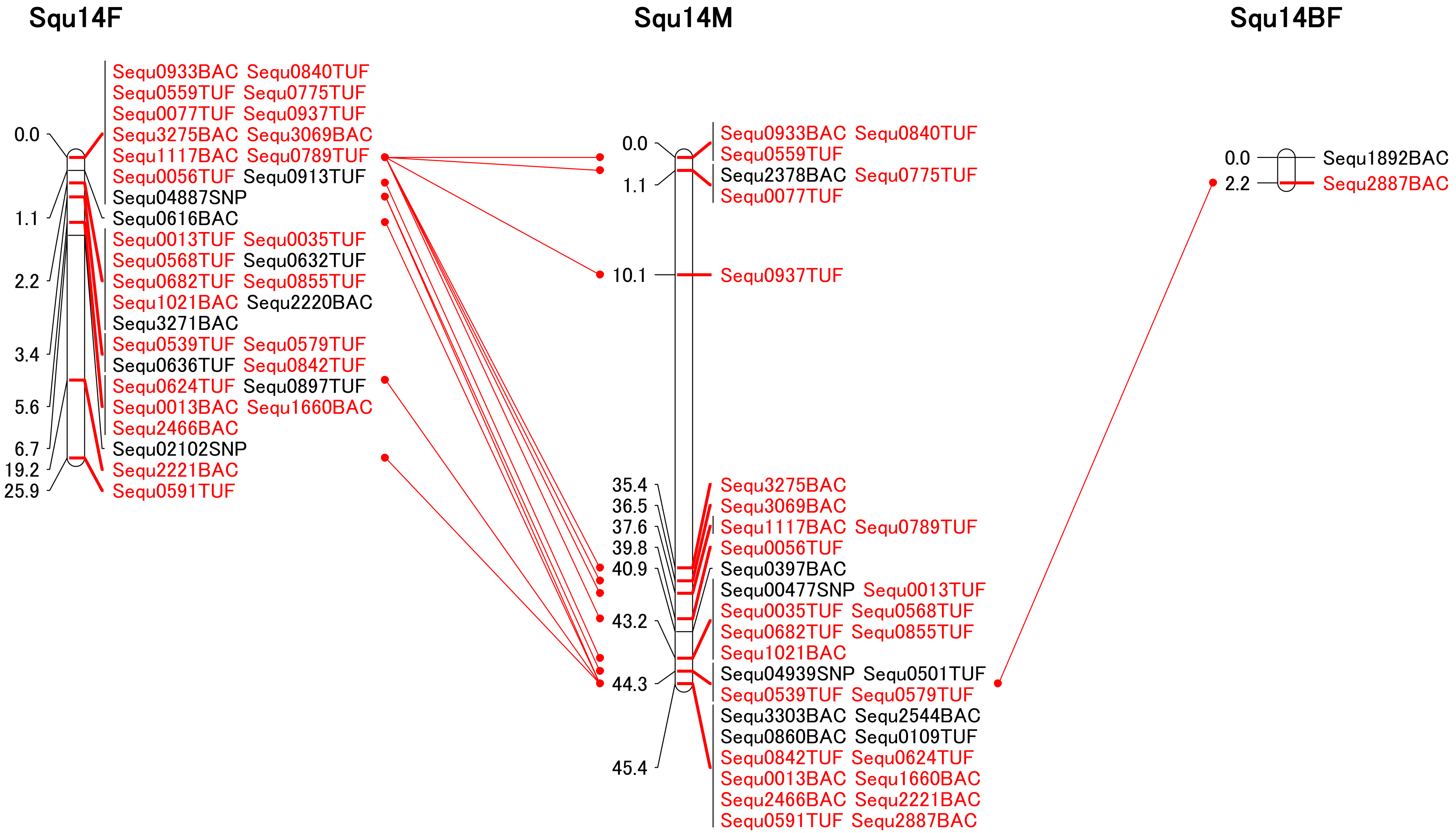
Squ14F
Squ14M
Squ14BF
Sequ0933BAC Sequ0840TUF
Sequ0559TUF Sequ0775TUF
Sequ0077TUF Sequ0937TUF
Sequ3275BAC Sequ3069BAC
Sequ1117BAC Sequ0789TUF
Sequ0056TUF Sequ0913TUF
Sequ04887SNP
Sequ0616BAC
Sequ0013TUF Sequ0035TUF
Sequ0568TUF Sequ0632TUF
Sequ0682TUF Sequ0855TUF
Sequ1021BAC Sequ2220BAC
Sequ3271BAC
Sequ0539TUF Sequ0579TUF
Sequ0636TUF Sequ0842TUF
Sequ0624TUF Sequ0897TUF
Sequ0013BAC Sequ1660BAC
Sequ2466BAC
Sequ02102SNP
Sequ2221BAC
Sequ0591TUF
0.0
1.1
2.2
3.4
5.6
6.7
19.2
25.9
0.0
1.1
10.1
35.4
36.5
37.6
39.8
40.9
43.2
44.3
45.4
Sequ0933BAC Sequ0840TUF
Sequ0559TUF
Sequ2378BAC Sequ0775TUF
Sequ0077TUF
Sequ0937TUF
Sequ3275BAC
Sequ3069BAC
Sequ1117BAC Sequ0789TUF
Sequ0056TUF
Sequ0397BAC
Sequ00477SNP Sequ0013TUF
Sequ0035TUF Sequ0568TUF
Sequ0682TUF Sequ0855TUF
Sequ1021BAC
Sequ04939SNP Sequ0501TUF
Sequ0539TUF Sequ0579TUF
Sequ3303BAC Sequ2544BAC
Sequ0860BAC Sequ0109TUF
Sequ0842TUF Sequ0624TUF
Sequ0013BAC Sequ1660BAC
Sequ2466BAC Sequ2221BAC
Sequ0591TUF Sequ2887BAC
0.0
2.2
Sequ1892BAC
Sequ2887BAC

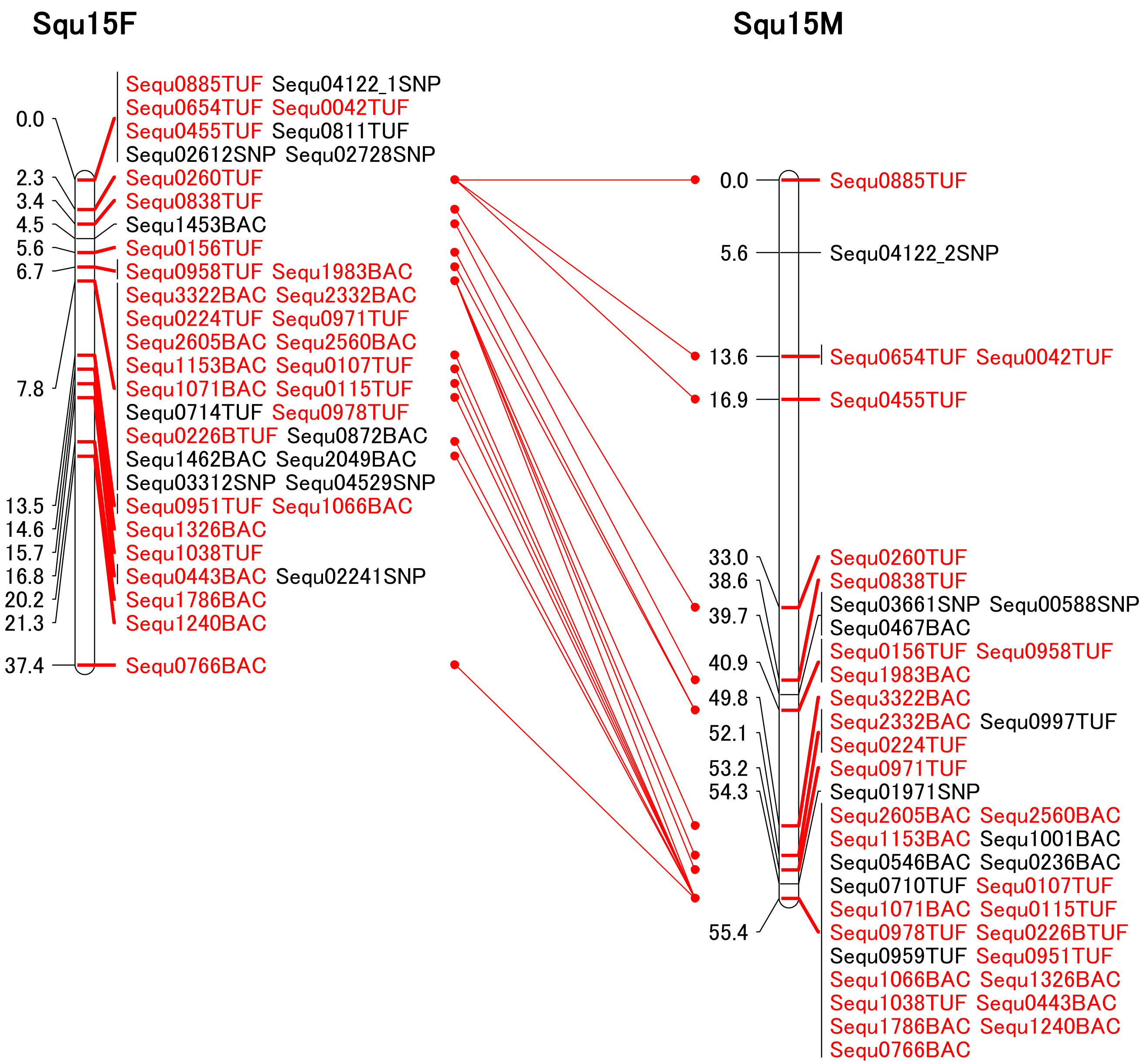
Squ15F
Squ15M
0.0
2.3
3.4
4.5
5.6
6.7
7.8
13.5
14.6
15.7
16.8
20.2
21.3
37.4
Sequ0885TUF Sequ04122_1SNP
Sequ0654TUF Sequ0042TUF
Sequ0455TUF Sequ0811TUF
Sequ02612SNP Sequ02728SNP
Sequ0260TUF
Sequ0838TUF
Sequ1453BAC
Sequ0156TUF
Sequ0958TUF Sequ1983BAC
Sequ3322BAC Sequ2332BAC
Sequ0224TUF Sequ0971TUF
Sequ2605BAC Sequ2560BAC
Sequ1153BAC Sequ0107TUF
Sequ1071BAC Sequ0115TUF
Sequ0714TUF Sequ0978TUF
Sequ0226BTUF Sequ0872BAC
Sequ1462BAC Sequ2049BAC
Sequ03312SNP Sequ04529SNP
Sequ0951TUF Sequ1066BAC
Sequ1326BAC
Sequ1038TUF
Sequ0443BAC Sequ02241SNP
Sequ1786BAC
Sequ1240BAC
Sequ0766BAC
0.0
5.6
13.6
16.9
33.0
38.6
39.7
40.9
49.8
52.1
53.2
54.3
55.4
Sequ0885TUF
Sequ04122_2SNP
Sequ0654TUF Sequ0042TUF
Sequ0455TUF
Sequ0260TUF
Sequ0838TUF
Sequ03661SNP Sequ00588SNP
Sequ0467BAC
Sequ0156TUF Sequ0958TUF
Sequ1983BAC
Sequ3322BAC
Sequ2332BAC Sequ0997TUF
Sequ0224TUF
Sequ0971TUF
Sequ01971SNP
Sequ2605BAC Sequ2560BAC
Sequ1153BAC Sequ1001BAC
Sequ0546BAC Sequ0236BAC
Sequ0710TUF Sequ0107TUF
Sequ1071BAC Sequ0115TUF
Sequ0978TUF Sequ0226BTUF
Sequ0959TUF Sequ0951TUF
Sequ1066BAC Sequ1326BAC
Sequ1038TUF Sequ0443BAC
Sequ1786BAC Sequ1240BAC
Sequ0766BAC

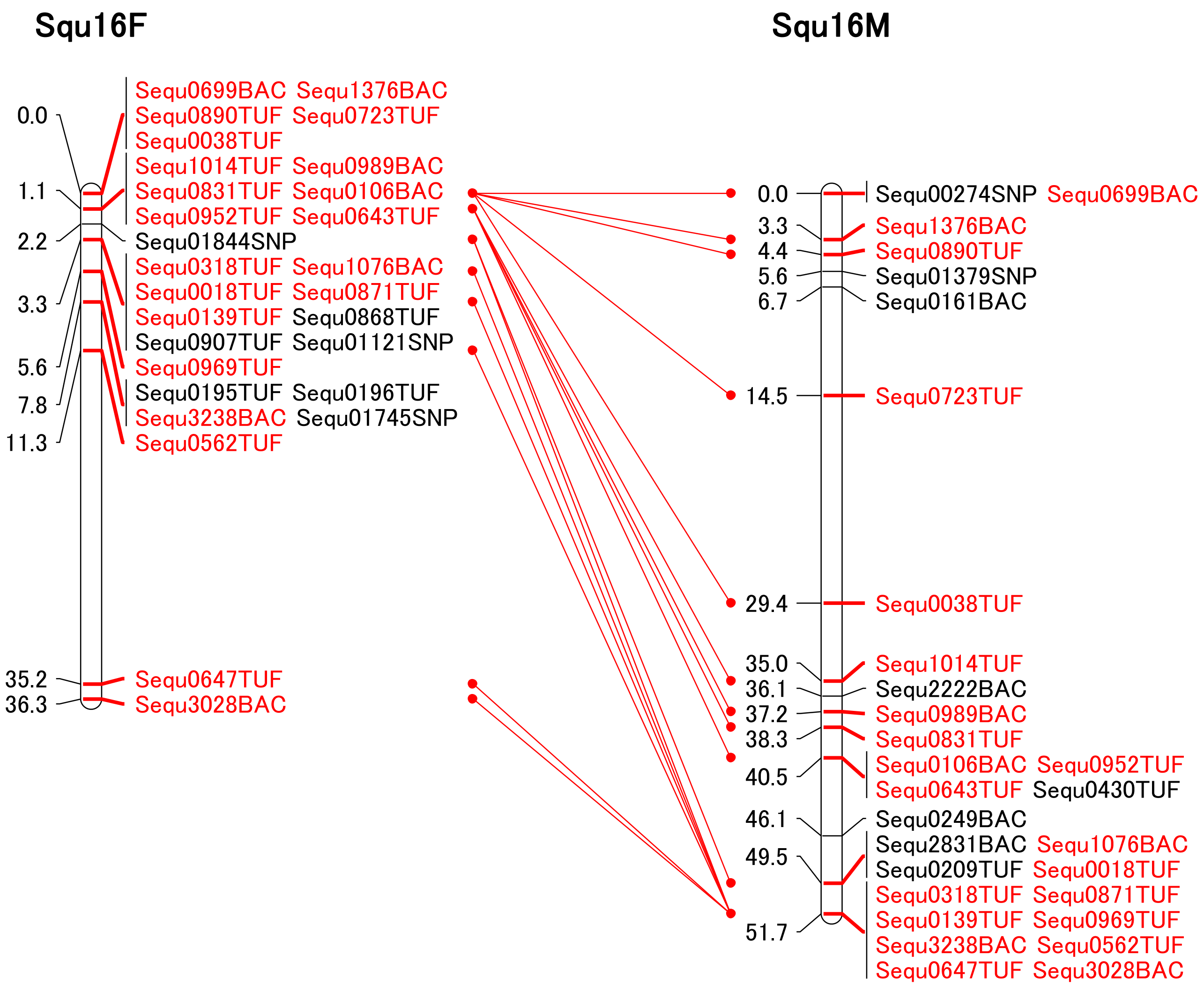

Squ16F
Squ16M
0.0
1.1
2.2
3.3
5.6
7.8
11.3
35.2
36.3
Sequ0699BAC Sequ1376BAC
Sequ0890TUF Sequ0723TUF
Sequ0038TUF
Sequ1014TUF Sequ0989BAC
Sequ0831TUF Sequ0106BAC
Sequ0952TUF Sequ0643TUF
Sequ01844SNP
Sequ0318TUF Sequ1076BAC
Sequ0018TUF Sequ0871TUF
Sequ0139TUF Sequ0868TUF
Sequ0907TUF Sequ01121SNP
Sequ0969TUF
Sequ0195TUF Sequ0196TUF
Sequ3238BAC Sequ01745SNP
Sequ0562TUF
Sequ0647TUF
Sequ3028BAC
0.0
3.3
4.4
5.6
6.7
14.5
29.4
35.0
36.1
37.2
38.3
40.5
46.1
49.5
51.7
Sequ00274SNP Sequ0699BAC
Sequ1376BAC
Sequ0890TUF
Sequ01379SNP
Sequ0161BAC
Sequ0723TUF
Sequ0038TUF
Sequ1014TUF
Sequ2222BAC
Sequ0989BAC
Sequ0831TUF
Sequ0106BAC Sequ0952TUF
Sequ0643TUF Sequ0430TUF
Sequ0249BAC
Sequ2831BAC Sequ1076BAC
Sequ0209TUF Sequ0018TUF
Sequ0318TUF Sequ0871TUF
Sequ0139TUF Sequ0969TUF
Sequ3238BAC Sequ0562TUF
Sequ0647TUF Sequ3028BAC

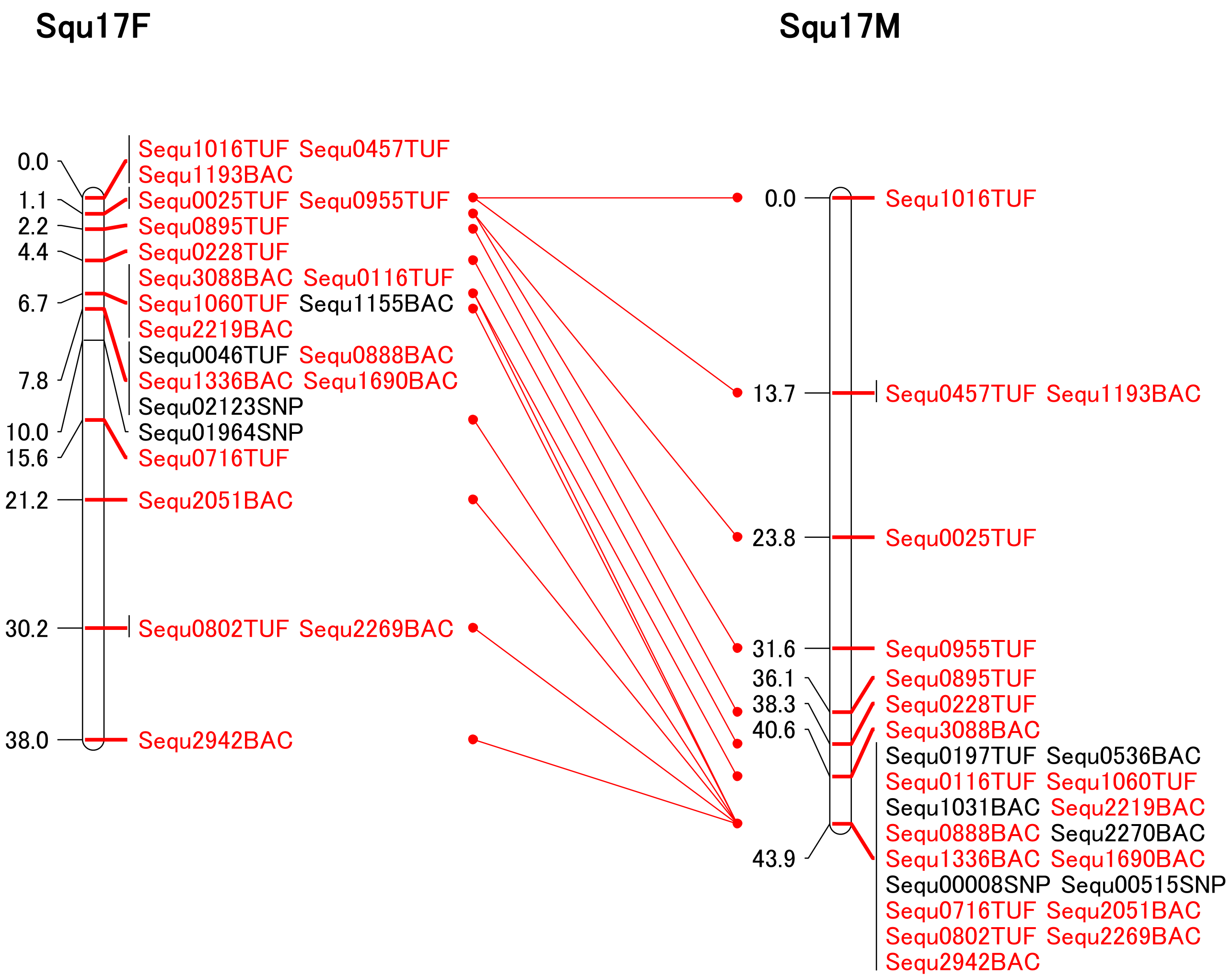

Squ17F
Squ17M
0.0
1.1
2.2
4.4
6.7
7.8
10.0
15.6
21.2
30.2
38.0
Sequ1016TUF Sequ0457TUF
Sequ1193BAC
Sequ0025TUF Sequ0955TUF
Sequ0895TUF
Sequ0228TUF
Sequ3088BAC Sequ0116TUF
Sequ1060TUF Sequ1155BAC
Sequ2219BAC
Sequ0046TUF Sequ0888BAC
Sequ1336BAC Sequ1690BAC
Sequ02123SNP
Sequ01964SNP
Sequ0716TUF
Sequ2051BAC
Sequ0802TUF Sequ2269BAC
Sequ2942BAC
0.0
13.7
23.8
31.6
36.1
38.3
40.6
43.9
Sequ1016TUF
Sequ0457TUF Sequ1193BAC
Sequ0025TUF
Sequ0955TUF
Sequ0895TUF
Sequ0228TUF
Sequ3088BAC
Sequ0197TUF Sequ0536BAC
Sequ0116TUF Sequ1060TUF
Sequ1031BAC Sequ2219BAC
Sequ0888BAC Sequ2270BAC
Sequ1336BAC Sequ1690BAC
Sequ00008SNP Sequ00515SNP
Sequ0716TUF Sequ2051BAC
Sequ0802TUF Sequ2269BAC
Sequ2942BAC

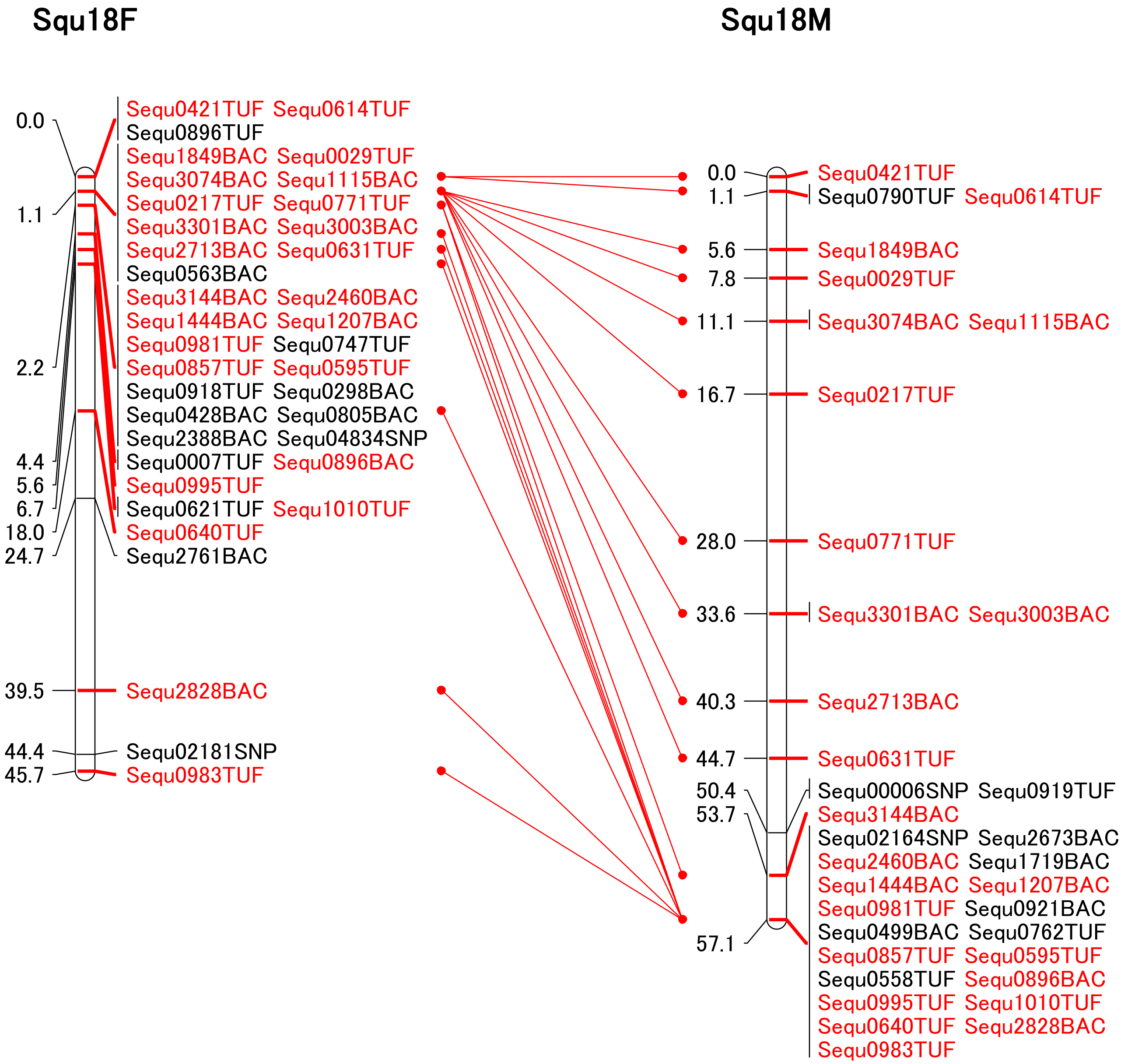
Squ18F
Squ18M
0.0
1.1
2.2
4.4
5.6
6.7
18.0
24.7
39.5
44.4
45.7
Sequ0421TUF Sequ0614TUF
Sequ0896TUF
Sequ1849BAC Sequ0029TUF
Sequ3074BAC Sequ1115BAC
Sequ0217TUF Sequ0771TUF
Sequ3301BAC Sequ3003BAC
Sequ2713BAC Sequ0631TUF
Sequ0563BAC
Sequ3144BAC Sequ2460BAC
Sequ1444BAC Sequ1207BAC
Sequ0981TUF Sequ0747TUF
Sequ0857TUF Sequ0595TUF
Sequ0918TUF Sequ0298BAC
Sequ0428BAC Sequ0805BAC
Sequ2388BAC Sequ04834SNP
Sequ0007TUF Sequ0896BAC
Sequ0995TUF
Sequ0621TUF Sequ1010TUF
Sequ0640TUF
Sequ2761BAC
Sequ2828BAC
Sequ02181SNP
Sequ0983TUF
0.0
1.1
5.6
7.8
11.1
16.7
28.0
33.6
40.3
44.7
50.4
53.7
57.1
Sequ0421TUF
Sequ0790TUF Sequ0614TUF
Sequ1849BAC
Sequ0029TUF
Sequ3074BAC Sequ1115BAC
Sequ0217TUF
Sequ0771TUF
Sequ3301BAC Sequ3003BAC
Sequ2713BAC
Sequ0631TUF
Sequ00006SNP Sequ0919TUF
Sequ3144BAC
Sequ02164SNP Sequ2673BAC
Sequ2460BAC Sequ1719BAC
Sequ1444BAC Sequ1207BAC
Sequ0981TUF Sequ0921BAC
Sequ0499BAC Sequ0762TUF
Sequ0857TUF Sequ0595TUF
Sequ0558TUF Sequ0896BAC
Sequ0995TUF Sequ1010TUF
Sequ0640TUF Sequ2828BAC
Sequ0983TUF

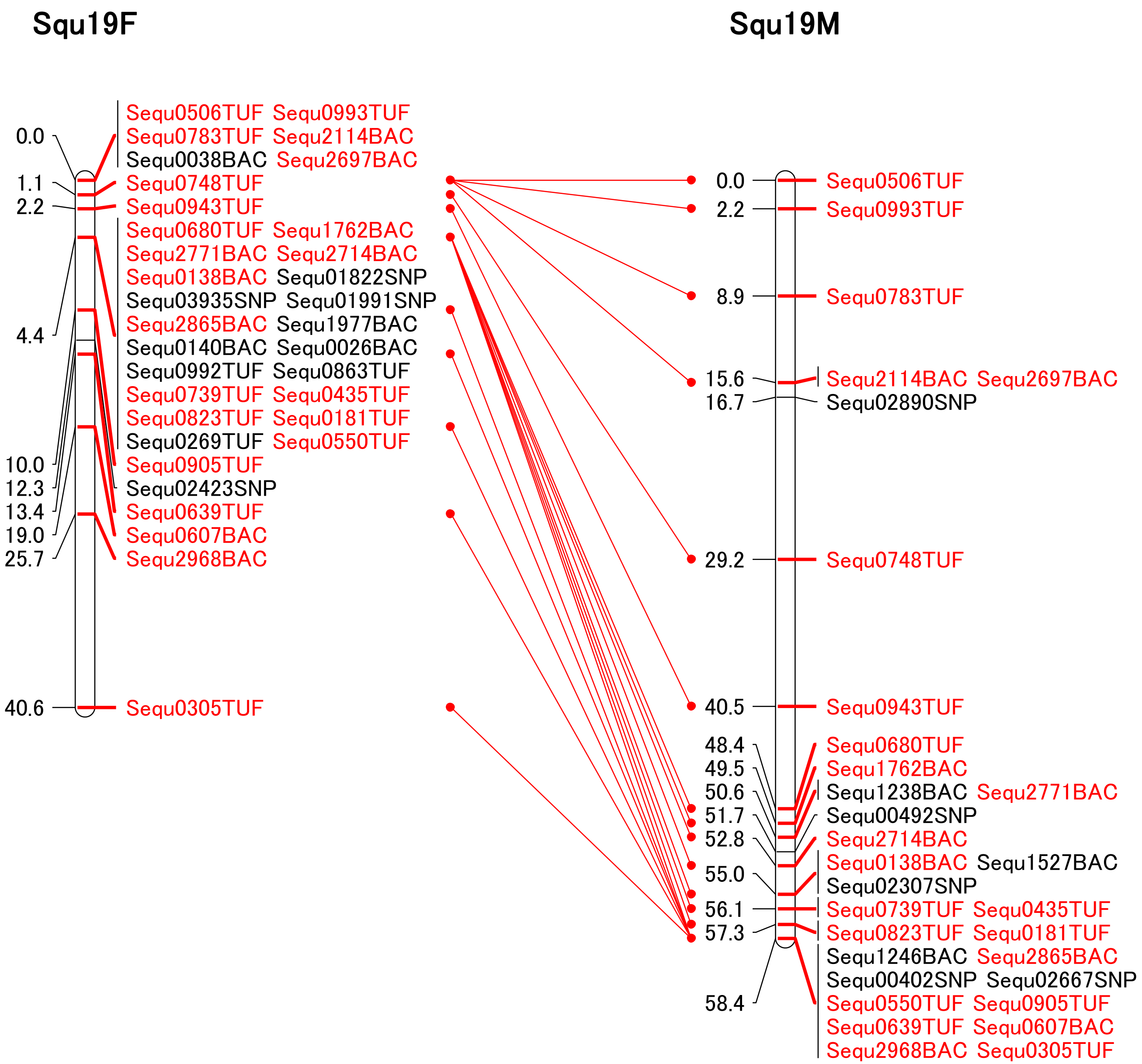

Squ19F
Squ19M
0.0
1.1
2.2
4.4
10.0
12.3
13.4
19.0
25.7
40.6
Sequ0506TUF Sequ0993TUF
Sequ0783TUF Sequ2114BAC
Sequ0038BAC Sequ2697BAC
Sequ0748TUF
Sequ0943TUF
Sequ0680TUF Sequ1762BAC
Sequ2771BAC Sequ2714BAC
Sequ0138BAC Sequ01822SNP
Sequ03935SNP Sequ01991SNP
Sequ2865BAC Sequ1977BAC
Sequ0140BAC Sequ0026BAC
Sequ0992TUF Sequ0863TUF
Sequ0739TUF Sequ0435TUF
Sequ0823TUF Sequ0181TUF
Sequ0269TUF Sequ0550TUF
Sequ0905TUF
Sequ02423SNP
Sequ0639TUF
Sequ0607BAC
Sequ2968BAC
Sequ0305TUF
0.0
2.2
8.9
15.6
16.7
29.2
40.5
48.4
49.5
50.6
51.7
52.8
55.0
56.1
57.3
58.4
Sequ0506TUF
Sequ0993TUF
Sequ0783TUF
Sequ2114BAC Sequ2697BAC
Sequ02890SNP
Sequ0748TUF
Sequ0943TUF
Sequ0680TUF
Sequ1762BAC
Sequ1238BAC Sequ2771BAC
Sequ00492SNP
Sequ2714BAC
Sequ0138BAC Sequ1527BAC
Sequ02307SNP
Sequ0739TUF Sequ0435TUF
Sequ0823TUF Sequ0181TUF
Sequ1246BAC Sequ2865BAC
Sequ00402SNP Sequ02667SNP
Sequ0550TUF Sequ0905TUF
Sequ0639TUF Sequ0607BAC
Sequ2968BAC Sequ0305TUF

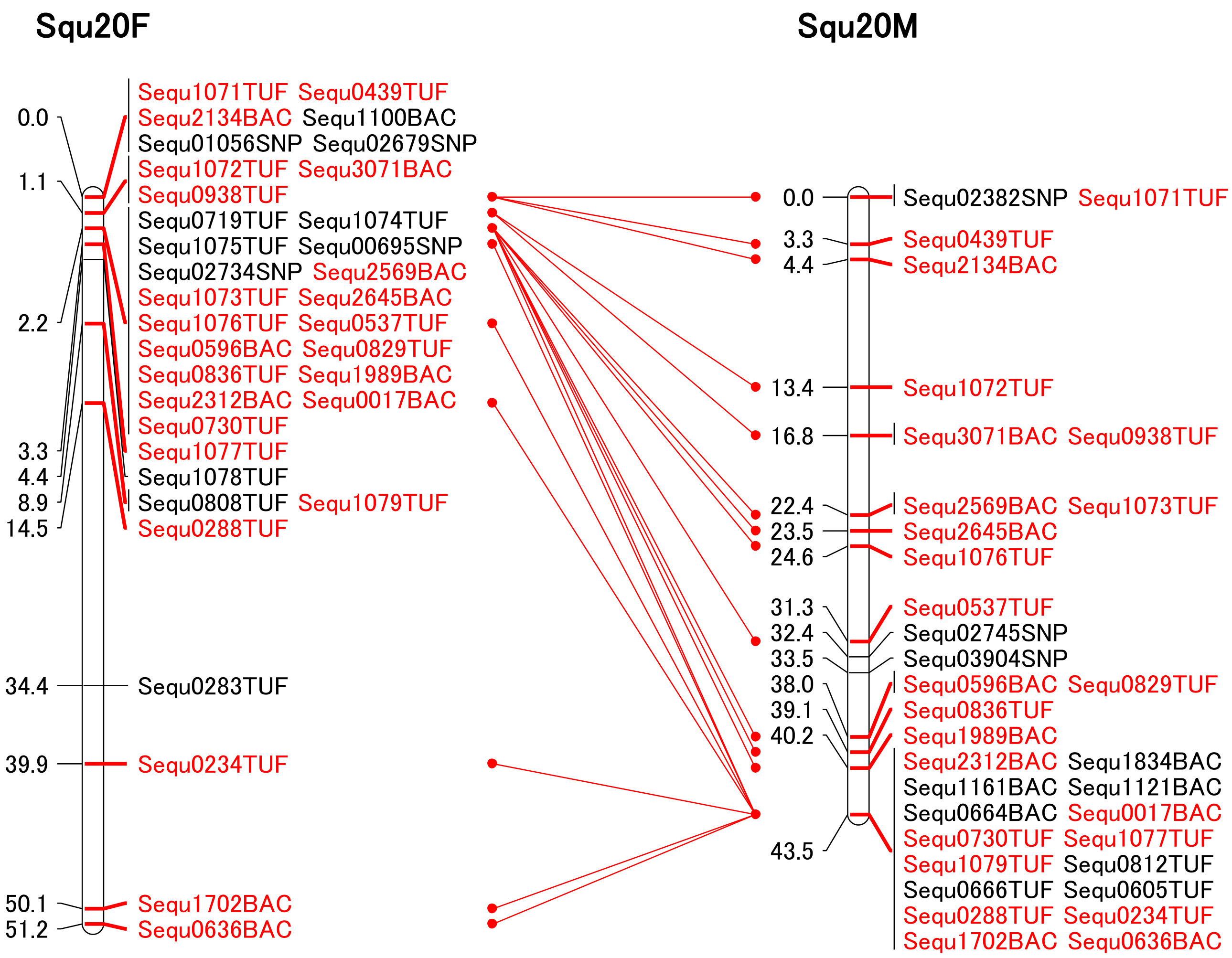
Squ20F
Squ20M
0.0
1.1
2.2
3.3
4.4
8.9
14.5
34.4
39.9
50.1
51.2
Sequ1071TUF Sequ0439TUF
Sequ2134BAC Sequ1100BAC
Sequ01056SNP Sequ02679SNP
Sequ1072TUF Sequ3071BAC
Sequ0938TUF
Sequ0719TUF Sequ1074TUF
Sequ1075TUF Sequ00695SNP
Sequ02734SNP Sequ2569BAC
Sequ1073TUF Sequ2645BAC
Sequ1076TUF Sequ0537TUF
Sequ0596BAC Sequ0829TUF
Sequ0836TUF Sequ1989BAC
Sequ2312BAC Sequ0017BAC
Sequ0730TUF
Sequ1077TUF
Sequ1078TUF
Sequ0808TUF Sequ1079TUF
Sequ0288TUF
Sequ0283TUF
Sequ0234TUF
Sequ1702BAC
Sequ0636BAC
0.0
3.3
4.4
13.4
16.8
22.4
23.5
24.6
31.3
32.4
33.5
38.0
39.1
40.2
43.5
Sequ02382SNP Sequ1071TUF
Sequ0439TUF
Sequ2134BAC
Sequ1072TUF
Sequ3071BAC Sequ0938TUF
Sequ2569BAC Sequ1073TUF
Sequ2645BAC
Sequ1076TUF
Sequ0537TUF
Sequ02745SNP
Sequ03904SNP
Sequ0596BAC Sequ0829TUF
Sequ0836TUF
Sequ1989BAC
Sequ2312BAC Sequ1834BAC
Sequ1161BAC Sequ1121BAC
Sequ0664BAC Sequ0017BAC
Sequ0730TUF Sequ1077TUF
Sequ1079TUF Sequ0812TUF
Sequ0666TUF Sequ0605TUF
Sequ0288TUF Sequ0234TUF
Sequ1702BAC Sequ0636BAC

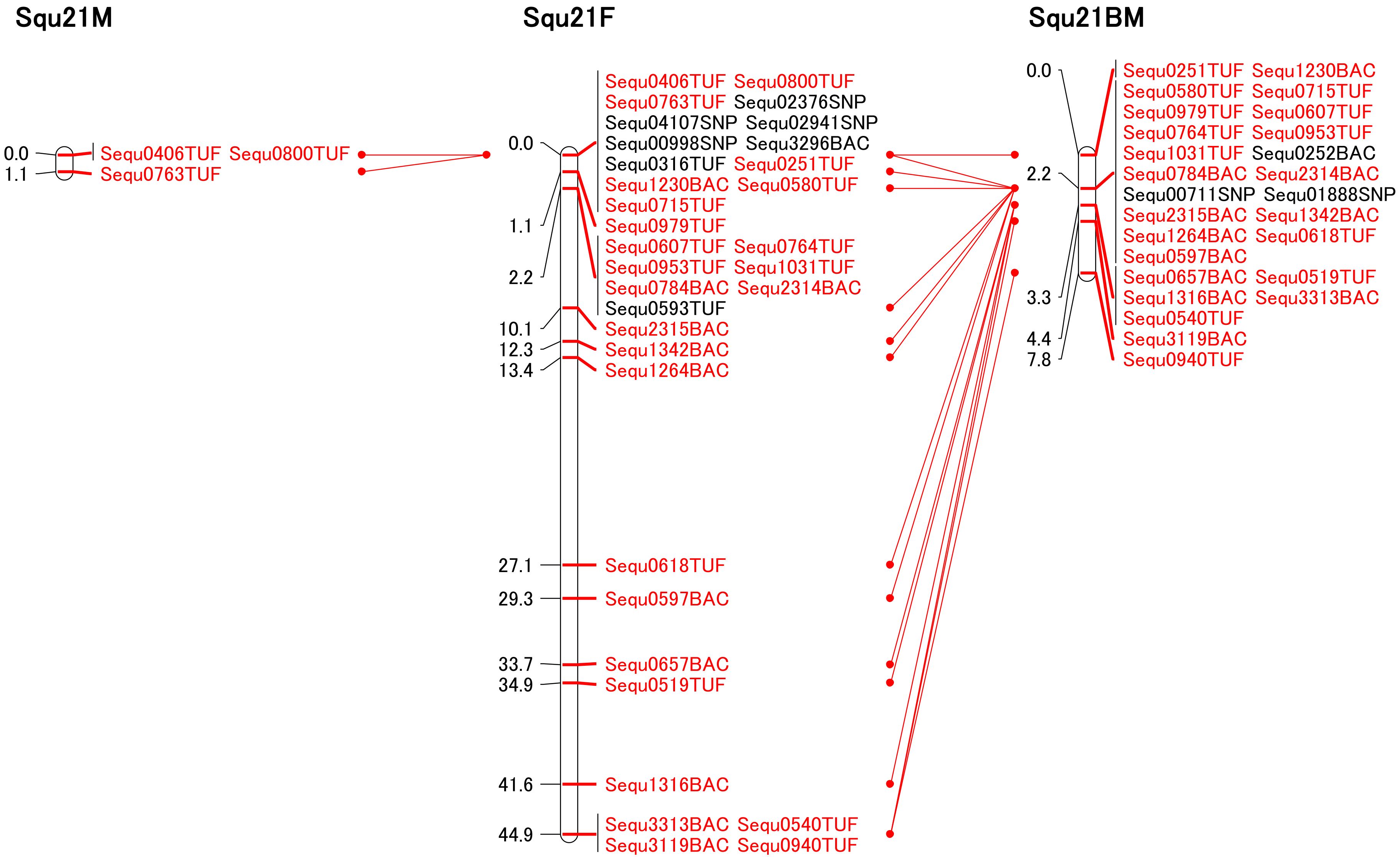

Squ21M
0.0
1.1
Sequ0406TUF Sequ0800TUF
Sequ0763TUF
Squ21F
0.0
1.1
2.2
10.1
12.3
13.4
27.1
29.3
33.7
34.9
41.6
44.9
Sequ0406TUF Sequ0800TUF
Sequ0763TUF Sequ02376SNP
Sequ04107SNP Sequ02941SNP
Sequ00998SNP Sequ3296BAC
Sequ0316TUF Sequ0251TUF
Sequ1230BAC Sequ0580TUF
Sequ0715TUF
Sequ0979TUF
Sequ0607TUF Sequ0764TUF
Sequ0953TUF Sequ1031TUF
Sequ0784BAC Sequ2314BAC
Sequ0593TUF
Sequ2315BAC
Sequ1342BAC
Sequ1264BAC
Sequ0618TUF
Sequ0597BAC
Sequ0657BAC
Sequ0519TUF
Sequ1316BAC
Sequ3313BAC Sequ0540TUF
Sequ3119BAC Sequ0940TUF
Squ21BM
0.0
2.2
3.3
4.4
7.8
Sequ0251TUF Sequ1230BAC
Sequ0580TUF Sequ0715TUF
Sequ0979TUF Sequ0607TUF
Sequ0764TUF Sequ0953TUF
Sequ1031TUF Sequ0252BAC
Sequ0784BAC Sequ2314BAC
Sequ00711SNP Sequ01888SNP
Sequ2315BAC Sequ1342BAC
Sequ1264BAC Sequ0618TUF
Sequ0597BAC
Sequ0657BAC Sequ0519TUF
Sequ1316BAC Sequ3313BAC
Sequ0540TUF
Sequ3119BAC
Sequ0940TUF

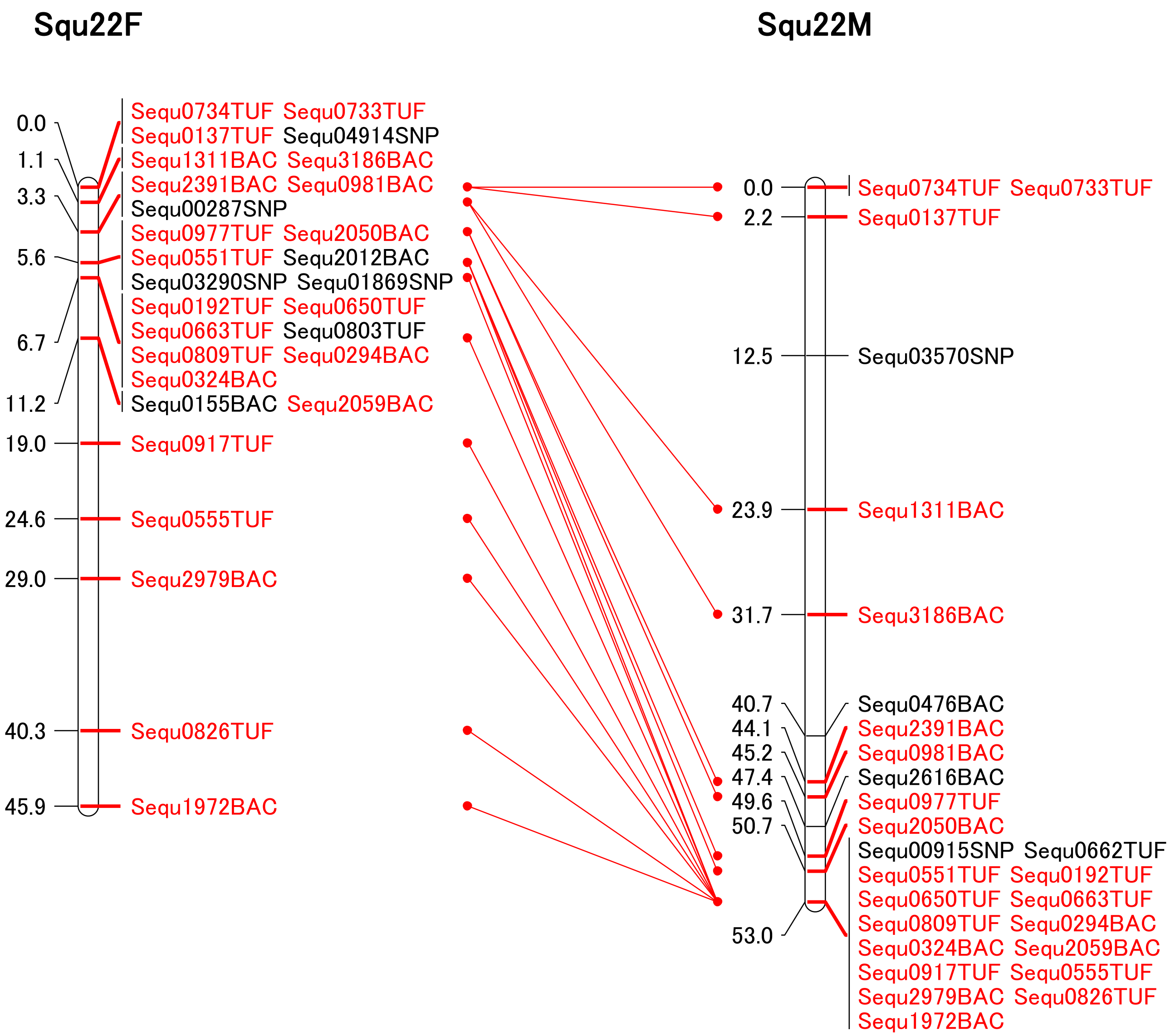
Squ22F
Squ22M
0.0
1.1
3.3
5.6
6.7
11.2
19.0
24.6
29.0
40.3
45.9
Sequ0734TUF Sequ0733TUF
Sequ0137TUF Sequ04914SNP
Sequ1311BAC Sequ3186BAC
Sequ2391BAC Sequ0981BAC
Sequ00287SNP
Sequ0977TUF Sequ2050BAC
Sequ0551TUF Sequ2012BAC
Sequ03290SNP Sequ01869SNP
Sequ0192TUF Sequ0650TUF
Sequ0663TUF Sequ0803TUF
Sequ0809TUF Sequ0294BAC
Sequ0324BAC
Sequ0155BAC Sequ2059BAC
Sequ0917TUF
Sequ0555TUF
Sequ2979BAC
Sequ0826TUF
Sequ1972BAC
0.0
2.2
12.5
23.9
31.7
40.7
44.1
45.2
47.4
49.6
50.7
53.0
Sequ0734TUF Sequ0733TUF
Sequ0137TUF
Sequ03570SNP
Sequ1311BAC
Sequ3186BAC
Sequ0476BAC
Sequ2391BAC
Sequ0981BAC
Sequ2616BAC
Sequ0977TUF
Sequ2050BAC
Sequ00915SNP Sequ0662TUF
Sequ0551TUF Sequ0192TUF
Sequ0650TUF Sequ0663TUF
Sequ0809TUF Sequ0294BAC
Sequ0324BAC Sequ2059BAC
Sequ0917TUF Sequ0555TUF
Sequ2979BAC Sequ0826TUF
Sequ1972BAC

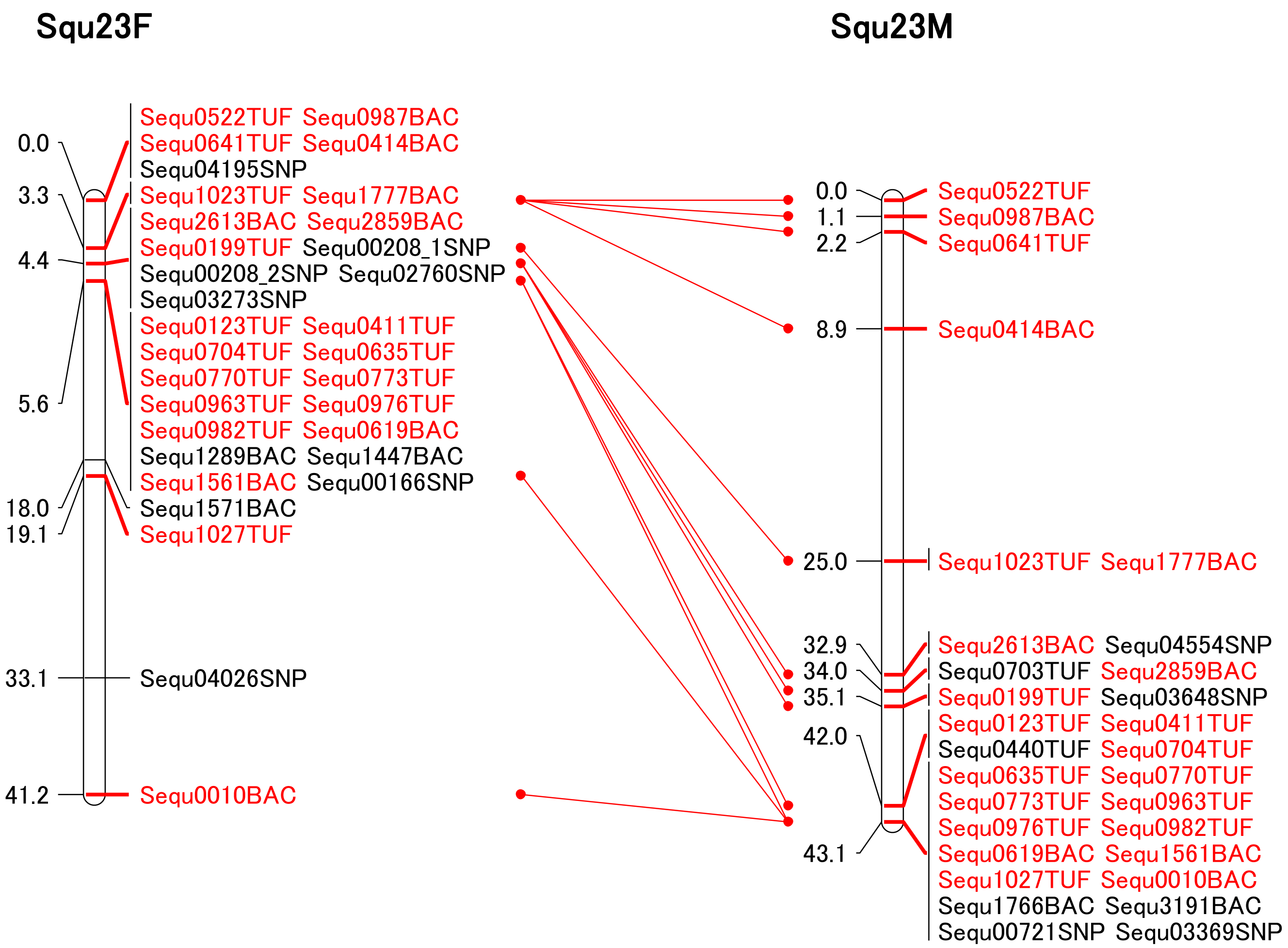

Squ23F
Squ23M
0.0
3.3
4.4
5.6
18.0
19.1
33.1
41.2
Sequ0522TUF Sequ0987BAC
Sequ0641TUF Sequ0414BAC
Sequ04195SNP
Sequ1023TUF Sequ1777BAC
Sequ2613BAC Sequ2859BAC
Sequ0199TUF Sequ00208_1SNP
Sequ00208_2SNP Sequ02760SNP
Sequ03273SNP
Sequ0123TUF Sequ0411TUF
Sequ0704TUF Sequ0635TUF
Sequ0770TUF Sequ0773TUF
Sequ0963TUF Sequ0976TUF
Sequ0982TUF Sequ0619BAC
Sequ1289BAC Sequ1447BAC
Sequ1561BAC Sequ00166SNP
Sequ1571BAC
Sequ1027TUF
Sequ04026SNP
Sequ0010BAC
0.0
1.1
2.2
8.9
25.0
32.9
34.0
35.1
42.0
43.1
Sequ0522TUF
Sequ0987BAC
Sequ0641TUF
Sequ0414BAC
Sequ1023TUF Sequ1777BAC
Sequ2613BAC Sequ04554SNP
Sequ0703TUF Sequ2859BAC
Sequ0199TUF Sequ03648SNP
Sequ0123TUF Sequ0411TUF
Sequ0440TUF Sequ0704TUF
Sequ0635TUF Sequ0770TUF
Sequ0773TUF Sequ0963TUF
Sequ0976TUF Sequ0982TUF
Sequ0619BAC Sequ1561BAC
Sequ1027TUF Sequ0010BAC
Sequ1766BAC Sequ3191BAC
Sequ00721SNP Sequ03369SNP

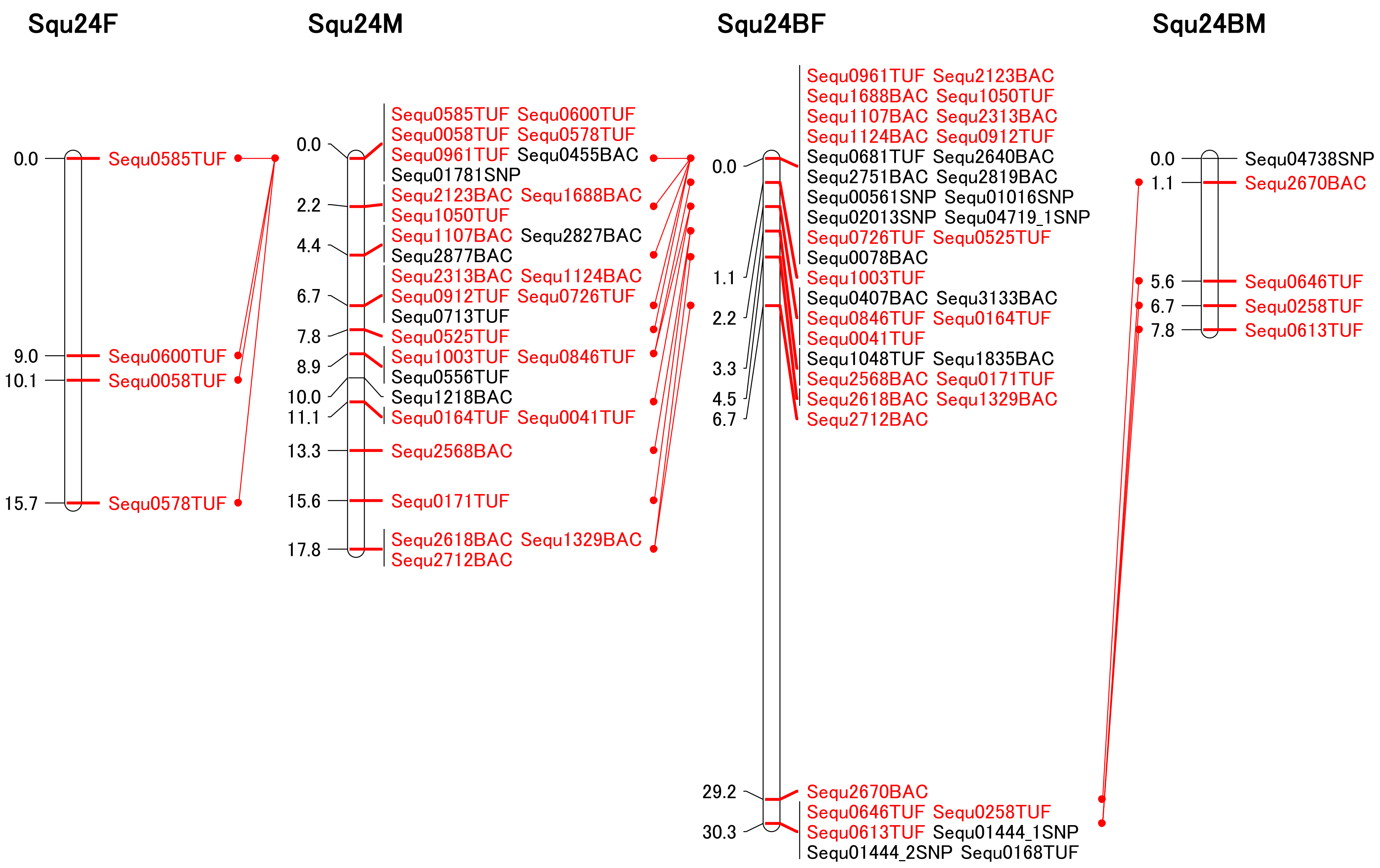
Squ24F
Squ24M
Squ24BF
Squ24BM
0.0 Sequ0585TUF
9.0 Sequ0600TUF
10.1 Sequ0058TUF
15.7 Sequ0578TUF
Sequ0585TUF Sequ0600TUF
Sequ0058TUF Sequ0578TUF
Sequ0961TUF Sequ0455BAC
Sequ01781SNP
0.0
2.2 Sequ2123BAC Sequ1688BAC
Sequ1050TUF
4.4 Sequ1107BAC Sequ2827BAC
Sequ2877BAC
6.7 Sequ2313BAC Sequ1124BAC
Sequ0912TUF Sequ0726TUF
Sequ0713TUF
7.8 Sequ0525TUF
8.9 Sequ1003TUF Sequ0846TUF
Sequ0556TUF
10.0 Sequ1218BAC
11.1 Sequ0164TUF Sequ0041TUF
13.3 Sequ2568BAC
15.6 Sequ0171TUF
17.8 Sequ2618BAC Sequ1329BAC
Sequ2712BAC
Sequ0961TUF Sequ2123BAC
Sequ1688BAC Sequ1050TUF
Sequ1107BAC Sequ2313BAC
Sequ1124BAC Sequ0912TUF
Sequ0681TUF Sequ2640BAC
Sequ2751BAC Sequ2819BAC
Sequ00561SNP Sequ01016SNP
Sequ02013SNP Sequ04719_1SNP
Sequ0726TUF Sequ0525TUF
Sequ0078BAC
Sequ1003TUF
Sequ0407BAC Sequ3133BAC
Sequ0846TUF Sequ0164TUF
Sequ0041TUF
Sequ1048TUF Sequ1835BAC
Sequ2568BAC Sequ0171TUF
Sequ2618BAC Sequ1329BAC
Sequ2712BAC
0.0
1.1
2.2
3.3
4.5
6.7
29.2 Sequ2670BAC
30.3 Sequ0646TUF Sequ0258TUF
Sequ0613TUF Sequ01444_1SNP
Sequ01444_2SNP Sequ0168TUF
0.0 Sequ04738SNP
1.1 Sequ2670BAC
5.6 Sequ0646TUF
6.7 Sequ0258TUF
7.8 Sequ0613TUF
